# Integrating hazard, exposure, vulnerability and resilience for risk and emergency management in a volcanic context: the ADVISE model

**DOI:** 10.1186/s13617-021-00108-5

**Published:** 2021-11-08

**Authors:** Costanza Bonadonna, Corine Frischknecht, Scira Menoni, Franco Romerio, Chris E. Gregg, Mauro Rosi, Sebastien Biass, Ali Asgary, Marco Pistolesi, Dehrick Guobadia, Alessandro Gattuso, Antonio Ricciardi, Chiara Cristiani

**Affiliations:** 1 Department of Earth Sciences, University of Geneva, Geneva, Switzerland; 2 Politecnico di Milano, Architettura e Pianificazione, Milan, Italy; 3 Geneva School of Economics and Management, University of Geneva, Geneva, Switzerland; 4 Institute for Environmental Sciences, Geneva, Switzerland; 5 Department of Geosciences, East Tennessee State University, Johnson City, USA; 6 Dipartimento di Scienze della Terra, Università Pisa, Pisa, Italy; 7 Earth Observatory of Singapore, Nanyang Technological University, Singapore, Singapore; 8 School of Administrative Studies, York University, Toronto, Canada; 9 Istituto Nazionale di Geofisica e Vulcanologia, Sezione Palermo - Sede operativa di Milazzo, Milazzo, Italy; 10 Dipartimento della Protezione Civile, Rome, Italy

**Keywords:** Risk assessment, Risk management, Emergency management, Hazard, Physical vulnerability, Functional vulnerability, Systemic vulnerability, Vulcano island

## Abstract

Risk assessments in volcanic contexts are complicated by the multi-hazard nature of both unrest and eruption phases, which frequently occur over a wide range of spatial and temporal scales. As an attempt to capture the multi-dimensional and dynamic nature of volcanic risk, we developed an *integrAteD VolcanIc risk asSEssment* (ADVISE) model that focuses on two temporal dimensions that authorities have to address in a volcanic context: short-term emergency management and long-term risk management. The output of risk assessment in the ADVISE model is expressed in terms of potential physical, functional, and systemic damage, determined by combining the available information on hazard, exposed systems and vulnerability. The ADVISE model permits qualitative, semi-quantitative and quantitative risk assessment depending on the final objective and on the available information. The proposed approach has evolved over a decade of study on the volcanic island of Vulcano (Italy), where recent signs of unrest combined with uncontrolled urban development and significant seasonal variations of exposed population result in highly dynamic volcanic risk. For the sake of illustration of all the steps of the ADVISE model, we focus here on the risk assessment of the transport system in relation to the tephra fallout associated with a long-lasting Vulcanian cycle.

**Supplementary Information:**

The online version contains supplementary material available at 10.1186/s13617-021-00108-5.

## Introduction

Developing methodologies to assess risk associated with natural hazards is an on-going challenge globally due to the complexity of assessing and combining the various risk factors (e.g. hazard, exposure, vulnerability, resilience) (e.g. Wisner et al. 2003). In particular, volcanic unrest and eruptions are potentially more diverse with respect to other natural hazards and pose significant threats to society on every continent. Regardless of the large international efforts to reduce risk, human fatalities are often still high (e.g. White Island 2019, New Zealand; Fuego 2018, Guatemala; Sinabung 2014, 2016, Indonesia; Ontake 2014, Japan; Merapi 2010, Indonesia). Large socio-economic impacts can also occur even when there are no casualties, such as when volcanic ash in the atmosphere affects the aviation industry (e.g. Eyjafjallajökull 2010, Iceland; Lund and Benediktsson 2011; Oxford Economics 2010)*.* Given the low frequency of high-impact volcanic eruptions, experiences in emergency planning and risk mitigation for eruptions remains limited. Associated impact and vulnerability studies also remain fragmented and unequally distributed amongst the different volcanic hazards, including primary (e.g. tephra fallout, pyroclastic density currents, lava flows, gas emissions) and secondary hazards (e.g. lahars, tsunamis, wind-induced remobilisation of pyroclastic deposits).

In order to mitigate risk and to enhance preparedness, a comprehensive risk assessment is required, although communities will certainly vary in the resources available to them for such purposes. The term risk refers to the expected loss as a function of hazard, exposure and vulnerability (e.g. UNDRO 1979; Fournier d'Albe 1979). *Hazard* is defined as a potentially damaging physical event, phenomenon or human activity, which may cause the loss of life or injury, property damage, social and economic disruption or environmental degradation, and characterised by its location, intensity, frequency and probability (ISDR 2004), whereas *exposed elements* is an inventory of people and artefacts exposed to the hazard, as well as their economic value; finally, *vulnerability* is the propensity to damage given intrinsic characteristics of people, assets and systems exposed. Whilst in the seventies most attention was devoted to the hazard component of risk, in the last decades new approaches have also included various aspects of vulnerability (e.g. Spence et al. 2005; Schneiderbauer and Ehrlich 2006; Menoni et al. 2012). In particular, since the 1980s, risk models have begun to better integrate vulnerability of people, assets and complex systems (e.g. Blong 1981; Weichselgartner and Bertens 2002; Birkmann 2006; Galoppin 2006; Wilson et al. 2017). As for resilience, while it had a variety of applications in the sciences and humanities (see Alexander (2013) for a review), it was only at the beginning of the twenty-first century that it has emerged on the international agenda with the Hyogo framework for action launched by the United Nations International Strategy for Disaster Risk Reduction (Manyena 2006). While strategies to assess hazard and exposure have now reached an acceptable level of consensus in the scientific community, the concepts of vulnerability and resilience remain a complex and hotly debated topic in disaster research (e.g. Coburn et al. 1991; Wisner et al. 2003; Cannon 2008; Norris et al. 2008; Li et al. 2010; Cutter 2013; Tiernan et al. 2019).

An integrated and holistic model of risk reduction assumes that all types of measures are considered, i.e. measures of prevention, mitigation, preparedness, response, recovery and reconstruction, and are equally applied. In such a model, the two fundamental activities of risk management and emergency management have to be correctly framed. Risk management acts in the long term to implement mitigation measures that are aimed at reducing damage and develop more resilient settlements and communities, whereas emergency management acts in the short term with the main objective of saving lives (e.g. Bonadonna et al. 2018). Long term and short term indicate here to involve time frames of years/decades and hours/days/weeks, respectively.

Regardless of the international effort towards the characterization of volcanic risk (e.g. Bonadonna et al. 2018), the integration of hazard and vulnerability assessments for a comprehensive evaluation of risk remains elusive. Pioneering works in quantitative risk assessment include the analyses of Spence et al. (2005), Biass et al. (2012, 2016a, 2016b, 2017), Scaini et al. (2014) and Thompson et al. (2016) for tephra fallout, Alberico et al. (2008) for pyroclastic density currents (PDCs), Bonne et al. (2008) and Favalli et al. (2009) for lava flows, and Lavigne (2000), Leung et al. (2003), and Mead and Magill (2017) for lahars. In addition, some examples of multi-hazard risk assessment also exist (e.g. Alberico et al. 2011; Alcorn et al. 2013; Neri et al. 2008; Pareschi et al. 2000; Zuccaro and Gregorio 2012). Nonetheless, most of these assessments describe the physical damage, as the socio-economic and systemic dimensions of vulnerability are more difficult to characterise. Therefore, matrix-based or indicators-based approaches are often applied when considering multi-dimensional vulnerabilities (e.g. van Westen and Greiving 2017). This leads to production of distinct and thematic risk maps based on potential physical damage and systemic consequences.

During the EU-supported project ENSURE (2008–2011), a new methodological framework for an integrated multi-scale vulnerability and resilience assessment across multiple temporal and spatial scales was developed (Enhancing resilience of communities and territories facing natural and na-tech hazards; Menoni et al. (2012)). A set of four-dimensional matrices for natural hazards (i.e. floods, landslides, volcanic eruptions, wildfires) identified aspects of vulnerability to consider before hazardous events occur. These matrices focused on the development of indicators of physical, functional, socio-economic and systemic vulnerability, while also considering the various ways to reduce vulnerability through adoption of certain protective actions or coping strategies that build resilience. This vision is largely integrated in the approach to risk and emergency management proposed herein as far as the vulnerability and resilience aspects are concerned. Nonetheless, a further proposed step is related to the identification of a method that combines the various components of the risk function (i.e. hazards, exposure, vulnerability and resilience) so as to obtain a risk assessment to be used as a basis for both risk and emergency management. Developing methodologies and models that assess risk as a combination of the factors described above is still challenging. As Simmons et al. (2017) suggest, if one wants to be exhaustive and consider impact to multiple assets and sectors, a purely quantitative approach is not really applicable, as it is inevitably limited to a few variables at a time (e.g. expected damage to buildings or to lifelines). A comprehensive approach therefore inevitably requires a mixed quantitative and qualitative approach (e.g. Eidsvig et al. 2017). Here we present the development of a new practical strategy and the results of its application for the compilation of risk assessment applied to the case study of La Fossa volcano (Vulcano, Italy). The model uses qualitative, semi-quantitative and quantitative approaches depending on the type and availability of data as well as on the final objective related to risk and emergency management.

## Methods

### The proposed *integrAteD VolcanIc risk asSEssment* (ADVISE) model

**Fig. 1:**
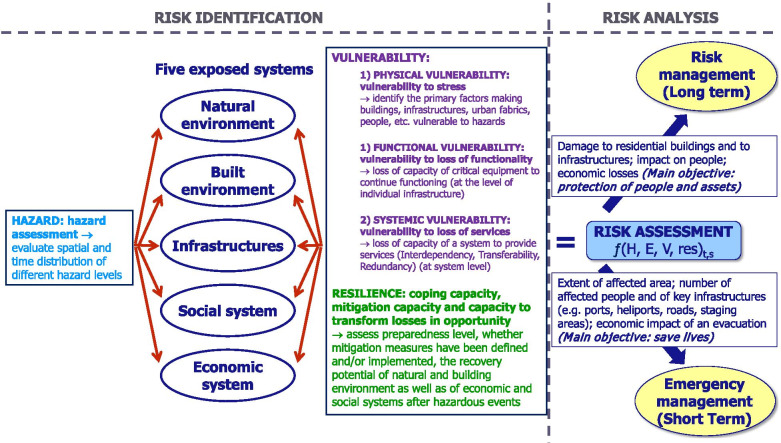
The methodological steps of the new Integrated Volcanic Risk Assessment (ADVISE) model

The proposed risk-assessment approach broadens the assessment of volcanic risk as defined by Fournier d'Albe (1979). In particular, risk assessment in the ADVISE model is a function of four aspects (hazard, exposure, vulnerability and resilience), the combination of which can be qualitative, semi-quantitative or quantitative depending on the availability of data and the approach adopted (Fig. 1). Physical and systemic vulnerabilities are the main dimensions characterised in the ADVISE framework, with functional vulnerability being an intrinsic aspect of systemic vulnerability. In this framework, social and economic vulnerability are not analysed per se but are integrated within both the exposure and vulnerability analyses (Fig. 1). The methodology that is described hereafter results from the blending of different steps that have been iteratively revised and recombined in the last years as part of the CERG-C program (Specialisation Certificate for the assessment and management of Geological and Climate Related Risk; http://www.unige.ch/sciences/terre/CERG-C/) and derived from the development of a number of European and national projects (e.g. ENSURE; Know4DRR; EDUCEN; IDEA). In the paper of Bonadonna et al. (2021) additional aspects of emergency management at Vulcano island are discussed (i.e. the efficiency assessment of an evacuation and the analysis of the potential economic impact of an evacuation).

### Framing long-term risk management and short-term emergency management within ADVISE

Long-term risk management is based on a risk analysis that identifies key areas where long-term mitigation actions could and should be implemented in order to reduce the consequences triggered by a volcanic event (Table 1). According to the proposed framework, the main goal of volcanic risk management is to reduce the vulnerability of people and structures, protect food and economic activities and maintain the functional capability of critical facilities and infrastructure by implementing both structural and non-structural measures (e.g. Blong 2013). In the natural hazard domain, structural measures are generally referring to engineering works to reduce the hazard extension or to reinforce structures, whilst the term non-structural mitigation covers a large spectrum of non-material measures such as educational and training activities and land-use planning (Bosher 2014).

**Table 1:** Main aspects to be assessed in the frame of long-term risk management

		Focus of analysis
**Hazard assessment**		➢ Time window (probability of occurrence in a certain period of time) ➢ Spatial extension ➢ Level of specific hazards and their variation with distance from source (e.g. concentration of gas, load of tephra, seismic ground acceleration)
**Exposure assessment**		➢ Identification and distribution of elements located within the area of potential hazard inundation: • buildings, roads, infrastructure (e.g. hospital, heliports, ports) • people • economic assets (e.g. agriculture, livestock, shops)
**Vulnerability assessment**	Physical dimension	➢ Selection of specific criteria to examine when considering the fragility of an element at risk towards a specific hazard and characterization of the behaviour depending on the level of hazard (fragility curves): • Residential buildings (typology defined based on the available parameters for hazard) • Infrastructure (e.g. power, water, telecommunication, road network) – analysis of potential weaknesses toward a specific hazard • People (e.g. day- and night-time distribution, composition of the population - residents, tourists, seasonal workers, age distribution, health status, literacy rate) • Agriculture (including livestock) – fragility analysis towards specific hazards • Shops/hotels/restaurants – fragility analysis towards specific hazards
	Systemic dimension (with focus on accessibility, redundancy, and interdependency)	➢ Accessibility to main facilities (e.g. health centre, school, heliport, harbours); this depends on the quality of the road network ➢ Accessibility to the island (e.g. availability of boats, weather conditions) ➢ Redundancy of infrastructure ➢ Interdependency between the Aeolian island, the main island (Sicily) and the mainland (Italy)
**Resilience assessment**		➢ Education in hazard and risk; mainstreaming of disaster risk management in various institutions (e.g. civil protection) ➢ Existence of structural (e.g. drainages for debris flows, roof reinforcement for tephra load) and non-structural (e.g. reconstruction plans, clean-up strategies, Master plans that account for potential hazards) mitigation measures ➢ Diversified economy
**Quantitative Risk Assessment**		➢ Number of buildings to be damaged, potential economic costs, potential quantity of debris generated (based on the number of buildings being damaged, considering their surface and height) ➢ Infrastructure to be damaged, economic cost, impact of the functionality loss on the community ➢ Number of people potentially being injured, killed, or affected ➢ Economic losses related to, for example, business interruption, loss of livestock, damage to crops, impact on tourism and disruption of transportation
**Complementary analysis to inform long-term risk management**		➢ Towards risk mitigation: • Land-use planning considering specific hazards: - Relocation of assets and infrastructures – risk avoidance - Protective measures – risk mitigation - Preparedness and contingency plans – risk control • Social aspects around these aspects (risk aversion, risk perception, participatory approaches) • Cost-benefit analysis of mitigation measures (structural / non-structural measures) ➢ Towards reconstruction: • Debris management • Reconstruction planning integrating lessons learned from damage assessment • Window of opportunity - alternative models of tourism and development for the island • Social aspects (participatory approach with different stakeholders) • Economic assessment of the different alternative models

Distinct from long-term risk management, emergency management mostly focuses on minimizing the number of fatalities and injured people (e.g. Merapi 2010, Indonesia; Mei and Lavigne 2013; Surono et al. 2011) (Table 2). This requires insights into specific dimensions of vulnerability such as physical vulnerability of the population, physical and systemic vulnerability of the transport system to guarantee accessibility to affected areas and to key emergency resources and personnel.

**Table 2:** Main aspects to be assessed in the frame of short-term emergency management

		Focus of analysis
**Hazard assessment**		➢ Spatial extension (boundaries) ➢ Level (for some hazard, e.g. only spatial extension is important in case of lava flows)
**Exposure assessment**		➢ Identification and distribution of elements located within the area of potential hazard inundation: • infrastructure key to evacuation (e.g. airports, ports, roads) • staging area for evacuation • people • livestock
**Vulnerability assessment**	Physical dimension	➢ Selection of specific criteria to examine when considering the fragility of an element at risk towards a specific hazard and characterization of the behaviour depending on the level of hazard (fragility curves): • Infrastructure key to evacuation (e.g. airports, ports, roads) • People (e.g. day- and night-time distribution, composition of the population - residents, tourists, seasonal workers, age distribution, health status, literacy rate)
	Systemic dimension (with focus on accessibility, redundancy, and interdependency)	➢ Accessibility to heliport and harbors (depends on the quality of the road network) ➢ Accessibility to the island (depends on weather conditions, state of the sea) ➢ Power, water and telecommunication failures (depend on how these infrastructure are affected by the considered hazard)
**Resilience assessment**		➢ Risk awareness; trust in authorities (e.g. civil protection); understanding of warning messages and compliance with required protective actions (e.g. mask, sheltering) ➢ Existence of early warning systems and evacuation plans
**Quantitative Risk Assessment**		➢ Extent of affected areas ➢ Number of affected infrastructure key to evacuation (e.g. airports, ports, roads) ➢ Estimated injured people and death toll in staging areas ➢ Estimated injured people and death toll in buildings ➢ Estimated impact on livestock
**Complementary analysis to inform emergency management**		➢ Analysis of potential economic impact of an evacuation (e.g. based on seasonality and differential areas to be evacuated) ➢ Efficiency assessment of evacuation (e.g. timing, wording of messages, circulation of information)

The first step for both emergency management and risk management is based on a comprehensive risk assessment. In order to assess the various factors summarised in Tables 1 and 2, the ADVISE model proposes a road map to assess each of the relevant factors that compose the risk function in a way to serve the two different purposes described above.

### Hazard assessment in the ADVISE model

The first step is the compilation of a hazard assessment (Fig. 1). With the term hazard we indicate the outcomes of a hazard assessment regardless of the approach used (i.e. deterministic, scenario-based or probabilistic hazard assessments). In order to be combined with the vulnerability maps, the final hazard outcome needs to describe the spatial distribution of hazard intensity (e.g., tephra ground accumulation, flow dynamic pressure) instead of the distribution of probability of a certain hazardous threshold (e.g. threshold of tephra load for roof collapse). Nonetheless, such a distribution of hazard intensity can be based on a probabilistic analysis addressing the epistemic and aleatoric uncertainties associated with the choice of eruptive scenarios (e.g. probabilistic isomass maps; Biass et al. 2012).

### Exposure assessment in the ADVISE model

The second step of the ADVISE model is the analysis of the distribution of exposed elements (i.e. exposure assessment) (Fig. 1). Exposure analyses consider a variety of sectors associated with a given area with the potential to be affected by a volcanic hazard, including the natural environment (e.g. flora, fauna, conservation areas), the built environment (e.g. residential houses, commercial buildings, critical infrastructure and facilities, public services), the social system (e.g. main characteristics of exposed people and institutions) and the economic system (including productive facilities). Exposure analysis mostly consists in the quantification of the number of assets pertaining to these systems that are located in hazardous areas and in the assessment of the associated economic value. The evaluation of exposure has tremendously improved thanks to the fast development of satellite and remote sensing technologies (Corbane et al. 2017). Estimation of economic value should be done for the tangibles (e.g. infrastructure, buildings, production of goods and services) as well as for the intangibles (e.g. population’s welfare, cultural heritage, environmental elements) values. These analyses depend on the data availability, and sometimes require alternative strategies (see following sections).

### Vulnerability assessment in the ADVISE model

The third step of the ADVISE model is the assessment of various dimensions of vulnerability (Fig. 1). First, the physical vulnerability of exposed assets is assessed, using, whenever possible, established models such as vulnerability curves (e.g. Spence et al. 2005; Zuccaro and Gregorio 2012; Blake et al. 2017a). The potential physical damage can either result from a quantitative characterization of both physical vulnerability and hazard, or through a qualitative evaluation of vulnerable elements. Either way, the potential physical damage is required for the consecutive analysis that includes both functional and systemic vulnerability. Systemic vulnerability aims at analyzing how urban and regional systems are able to respond to the physical damage triggered by a hazardous event. The methodology for assessing systemic vulnerability proposed by ADVISE builds on the ENSURE framework (Menoni et al. 2012) where, following Van der Veen and Logtmeijer (2005), systemic vulnerability is considered as a function of interdependency, redundancy and transferability. Interdependency looks at how the various systems are interconnected one to the other in complex environments such as cities and regions. This includes, for example, reliance on the power network (e.g. Rinaldi et al. 2001; Wardman et al. 2012) or on communication systems. Interdependencies in economic systems are also very important, as the lack of an intermediate input (e.g., due to the destruction of a factory) can disrupt the entire production chain of a certain good, which can potentially cascade into negative impact on export and, eventually, gross domestic product (GDP). Redundancy refers to alternative assets that provide the same service, while transferability refers to the possibility that one function or service can be relocated in another place in case of need (even if temporarily).

Regarding infrastructure, internal relations and hierarchy among components and parts can be referred to as functional vulnerability, the lack of capacity of a system to keep functioning during an event (Menoni et al. 2007). Besides functional aspects intrinsic to each complex system such as transportation or power networks, systemic vulnerability also considers fragilities deriving from the interconnections among different systems (such as between lifelines or between lifelines and other services and facilities that depend on them).

### Resilience concept in the ADVISE model

Resilience is a positive property that is more than just the counter part of vulnerability; it is, in fact, a complex concept that finds many definitions in the disaster risk literature (e.g. Manyena 2006; Cutter et al. 2008; UNISDR 2009; CARRI 2013; Atreya and Kunreuther 2020), but with rather few applications in volcanic risk (e.g. MIAVITA 2012). In the ADVISE model, resilience includes both coping capacities require to face disasters (short-term) and adaptative capacities (long-term) necessary to adjust to hazards (Frischknecht et al. 2010; ENSURE 2011). Therefore, in ADVISE we look at characteristics of systems that enhance the capacity to deal with and recover from events as well as to mitigate risk, including the existence of early-warning systems, implementation of mitigation measures, risk awareness, contingency planning. In that sense, we are in line with the definition provided by UNDRR (2017; https://www.undrr.org/terminology), i.e. *the ability of a system, community or society exposed to hazards to resist, absorb, accommodate, adapt to, transform and recover from the effects of a hazard in a timely and efficient manner, including through the preservation and restoration of its essential basic structures and functions through risk management*.

## Results

### Case study: the island of Vulcano

**Fig. 2:**
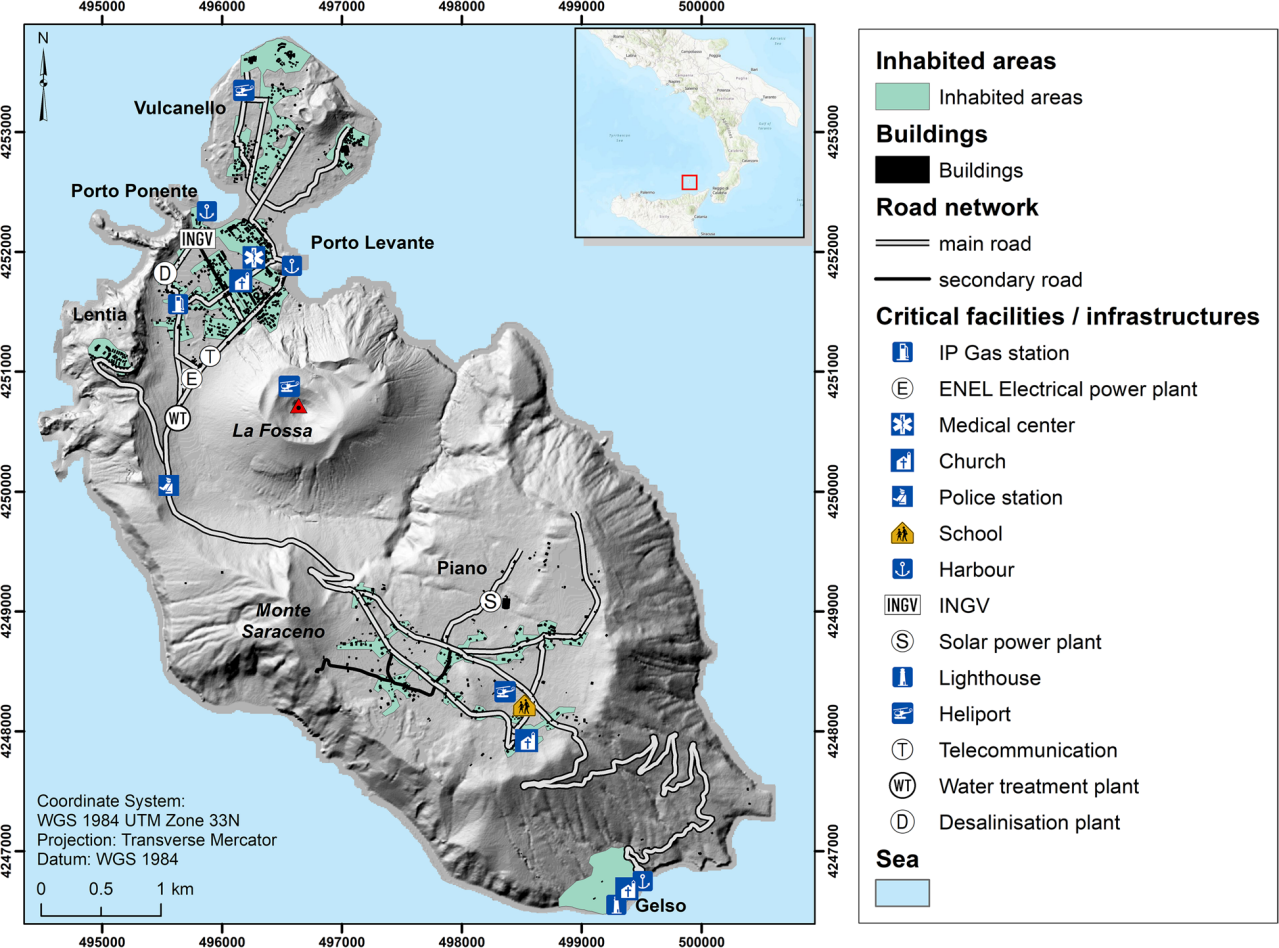
Geographic location of Vulcano island and description of the main inhabited centres (green areas), building distribution (black areas; adapted from Galderisi et al. 2013) and infrastructure and facilities

Vulcano is one of the seven islands of the Aeolian archipelago in the Tyrrhenian Sea of Southern Italy, having a surface area of 20 km^2^ and population of 1282 permanent residents (Fig. 2). The principal activity on the island until the last eruption of La Fossa volcano in 1888–1890 was harvesting wood and grapes and mining alum and sulphur. As it was the case in the other Aeolian Islands, tourism became the primary economic activity in the 1950s, but the main urbanization wave on Vulcano took place in the 1980s. Since then, development has progressed rapidly without consideration of volcanic hazards in land-use planning. There are three distinctly different populations of people at risk on Vulcano: i) the resident population, ii) seasonal migrant workers who support the summer visitor population and construction activities in the Spring to prepare for high season, and iii) visitors, whom, collectively speaking, represent a wide range of ethnic groups and demographics, speaking a host of languages. In particular, the seasonal migrant workers and visitors represent a substantial challenge to effectively respond to warnings of an eruption. Three main seasons have been identified on Vulcano: Low Season (November–April) with no touristic activity on the island, Middle Season (April–May-June and September–October) with a gradual increase in number of tourists, and High Season (July–August) when the island’s population swells to as many as about 22,000–28,000 per month (Tables 3 and 4), although most of these visitors do not stay overnight on the island. About two-thirds (67%) of the residents live in the areas of Porto Levante and Vulcanello in the north end of the island, 30% lives in Piano located in the South of the island, and 3% is distributed in two small area: Lentia, a residential subdivision on the west central side of the island, and Gelso, a more remote and scattered community in the far south of the island (Comune di Lipari 2017 personal communication). Most tourist infrastructure are located in the area of Porto Levante (known locally as *Porto)*, beneath the lowest flank of La Fossa cone (Fig. 2), the most active volcanic system on the island at present time. The seasonal variation of population size significantly increases the volcanic risk in the summer months. Critical facilities are distributed between Porto and Piano area (Fig. 2), resulting in a relatively complex territorial vulnerability associated with different eruptive scenarios. The potential for short warning times and high density of people and assets in some exposed areas exacerbate the risk to people and property. For example, the eruption in 1888–1890 occurred without any warning (although without the monitoring capabilities available today), and when the number of residents was significantly smaller than today. In addition, volcanic regions that represent a tourist attraction and where tourists are allowed to go very close to the hazardous areas are especially at risk (e.g., 2014 eruption of Ontake, Japan (Oikawa et al. 2016); 2019 eruption of White Island, New Zealand (Lim and Flaherty 2020)).

**Table 3:** Tourists arriving to Vulcano and staying in Vulcano for at least one night based on data for 2017 from the Osservatorio Turistico dell’Assessorato Turismo della Regione Sicilia. Presence on the island is calculated by multiplying the number of arrivals by the number of nights spent in hotel on the island

	Arrivals		Presence on the island		TOTAL Presence on the island
	Italians	Foreigners	Italians	Foreigners	
January	427	388	1255	996	2251
February	427	388	1255	996	2251
March	427	388	1255	996	2251
April	427	388	1255.4	996	2251
May	427	388	1255.4	996	2251
June	3511	1257	12,099	4532	16,631
July	4200	1836	15,638	6423	22,061
August	5511	1391	23,061	4901	27,962
September	2787	1831	12,232	6412	18,644
October	121	284	557	688	1245
November	121	284	557	688	1245
December	121	284	557	688	1245
Total	18,509	9106.8	70,979	29,310	100,289

**Table 4:** Tourists arrived to Vulcano from Milazzo and from the other Aeolian islands based on the main ferry companies and mini-cruises (data from Milazzo Port Authority for 2017). Mini-cruises arrive to Vulcano only during the summer months. Important to consider that in these numbers, trips of residents are also included

	Liberty Lines	Siremar	Mini-Cruisers (various companies)	TOTAL	Total daily arrivals
January	3996	356	–	4352	140
February	5623	426	–	6049	216
March	7222	785	–	8007	258
April	16,211	2265	4714	23,190	773
May	17,942	2680	7123	27,745	895
June	24,106	5030	9855	38,991	1300
July	30,935	7903	12,837	51,675	1667
August	41,146	13,857	22,377	77,380	2496
September	21,695	4552	10,670	36,917	1230
October	14,165	1772	–	15,937	514
November	6188	748	–	6936	231
December	3874	627	–	4501	145
Total	193,103	41,001	67,576	301,680	826

The island consists of four, juxtaposed volcanic edifices (Vulcano Primordiale, Lentia, La Fossa cone and Vulcanello) and two calderas (Caldera del Piano and Caldera La Fossa) (Zanella et al. 2001; De Astis et al. 2013) (Fig. 2). We focus our study on the activity of La Fossa cone, which represents the current most active volcanic system on the island. La Fossa is a 391 m-high quiescent volcanic cone that first erupted ca. 5.5 ka ago (Frazzetta et al. 1984). The last 1000 years of activity have been characterised by a large variety of eruptive styles and magma compositions, including effusive eruptions (e.g. AD 1739 Pietre Cotte lava flow), Vulcanian cycles (e.g. AD 1888–1890 eruption described by Mercalli and Silvestri (1891)) and higher intensity, short-lived events. Among the latter, two suplinian events (Pal B and Pal D eruptions of Di Traglia et al. 2013) occurred during AD XI-XII centuries, being bracketed by Vulcanello lavas and Breccia di Commenda deposits (Di Traglia et al. 2013; Fusillo et al. 2015). La Fossa activity ended with the products of the AD 1888–1890 Vulcanian eruption, mostly consisting of trachytic and rhyolitic ash and lapilli layers and breadcrust bombs. ﻿This eruptive cycle lasted 19 months and is by far the best described Vulcanian event occurred at Vulcano (Mercalli and Silvestri 1891), a milestone that provides information about dynamics, timing, products, and hazard. The activity was characterised by intermittent activity with convective columns with height up to 10 km, significant ballistics ejection, abundant gas and steam emissions, and repose time for single explosions from 4 to 72 h (Selva et al. 2020).

A large volume of the loose pyroclastic material produced during the activity of the last 1000 years has been largely remobilised during the rainy seasons generating frequent lahars that have largely inundated the North of the island up to the beach of Baia di Levante (e.g. Baumann et al. 2019). Historical chronicles (Mercalli and Silvestri 1891; De Fiore 1922), archeomagnetic data (Arrighi et al. 2006; Zanella et al. 1999; Zanella et al. 2001) and stratigraphic investigations (Di Traglia et al. 2013; De Astis et al. 1997, 2013) concur in indicating that the number of effusive and explosive eruptions that have occurred in the past 1000 years ranges between 15 and 23 (Selva et al. 2020). Since the end of the last magmatic eruption in 1888–90, the activity at La Fossa cone has mainly consisted of fumarolic emissions, ground deformation, earthquakes, and accompanying landslides (these pose a threat of small, localized tsunamis) (Barberi et al. 1991). Fumarolic fluids are discharged almost totally in two main fumarolic fields located in the northern rim of the active crater of La Fossa cone and at the beach of Baia di Levante (Porto area; Fig. 2). Two major episodes of volcanic unrest have occurred since the magmatic eruption of 1888–1890 which were characterised by several fluctuations in fumaroles temperature and in their chemical composition (1913–1923 and 1977 to present; e.g. Granieri et al. 2006; Sicardi 1940). Between March and June 1988, a period of high regional seismic activity resulted in a landslide in April 1988, collapsing a part of the eastern coastal side of La Fossa cone into the sea. Following the latter episodes of activity in the late twentieth Century, increased scientific monitoring of volcanic activity was financed by the Italian Civil Protection Department. This led to detection of a phase of significant ground deformation on Vulcano in 1990. During the last 40 years La Fossa crater was also affected by discrete events of local anomalous seismicity coinciding with peaks of CO_2_ emissions (e.g. 1985, 1988, 1996, 2004, 2005; Granieri et al. 2006). Given these signs of volcanic unrest combined with a complex vulnerability of the island due to uncontrolled urban development and significant seasonal variation of the exposed population, the Italian Civil Protection Department has financed detailed studies on the potential eruptive scenarios that threaten the island (Selva et al. 2020).

### Hazard analysis

Potential eruptive hazards associated with an eruption of La Fossa volcano include both primary (e.g. increased gas emissions, tephra fallout, PDCs, lava flows; e.g. Frazzetta et al. 1984; Dellino et al. 2011; Biass et al. 2016a, 2016b; Granieri et al. 2014) and secondary hazards (e.g. lahars, landslides, tsunami; e.g. Ferrucci et al. 2005; Tinti et al. 1999; Baumann et al. 2019) (see a review by Selva et al. 2020). A comprehensive risk analysis should include all possible hazards and the interconnection between primary and secondary hazards (e.g. Baumann et al. 2019). However, for purposes of illustration, here we consider tephra fallout only, primarily because: i) it was the main hazard associated with the 1888–90 Vulcanian cycle, ii) it could also be associated with subplinian eruptions as observed in the stratigraphic record (e.g. Pal B and Pal D eruptions of Di Traglia et al. 2013), and iii) because it is considered one of the most likely hazards associated with future activity on Vulcano (Selva et al. 2020).

The hazard assessment is subdivided into two steps: event analysis involving evaluation of the eruption scenarios and their probability of occurrence, and extension analysis involving evaluation of the extent of the threat. The event analysis is mainly based on the stratigraphic record at La Fossa, where Vulcanian cycles are associated with the highest recurrence rate, i.e. at least five long-lasting episodes in the last 1000 years corresponding to annual frequency of 5 × 10^− 3^ year^− 1^, followed by rarer phreatic eruptions (2-3 × 10^− 3^ year^− 1^), subplinian/sustained events (1-3 × 10^− 3^ year^− 1^) and Strombolian explosions (1-3 × 10^− 3^ year^− 1^) (Selva et al. 2020). The extension analysis is typically carried out with empirical, analytical and/or numerical models as described below for the case study of tephra fallout.

#### Tephra fallout

**Fig. 3:**
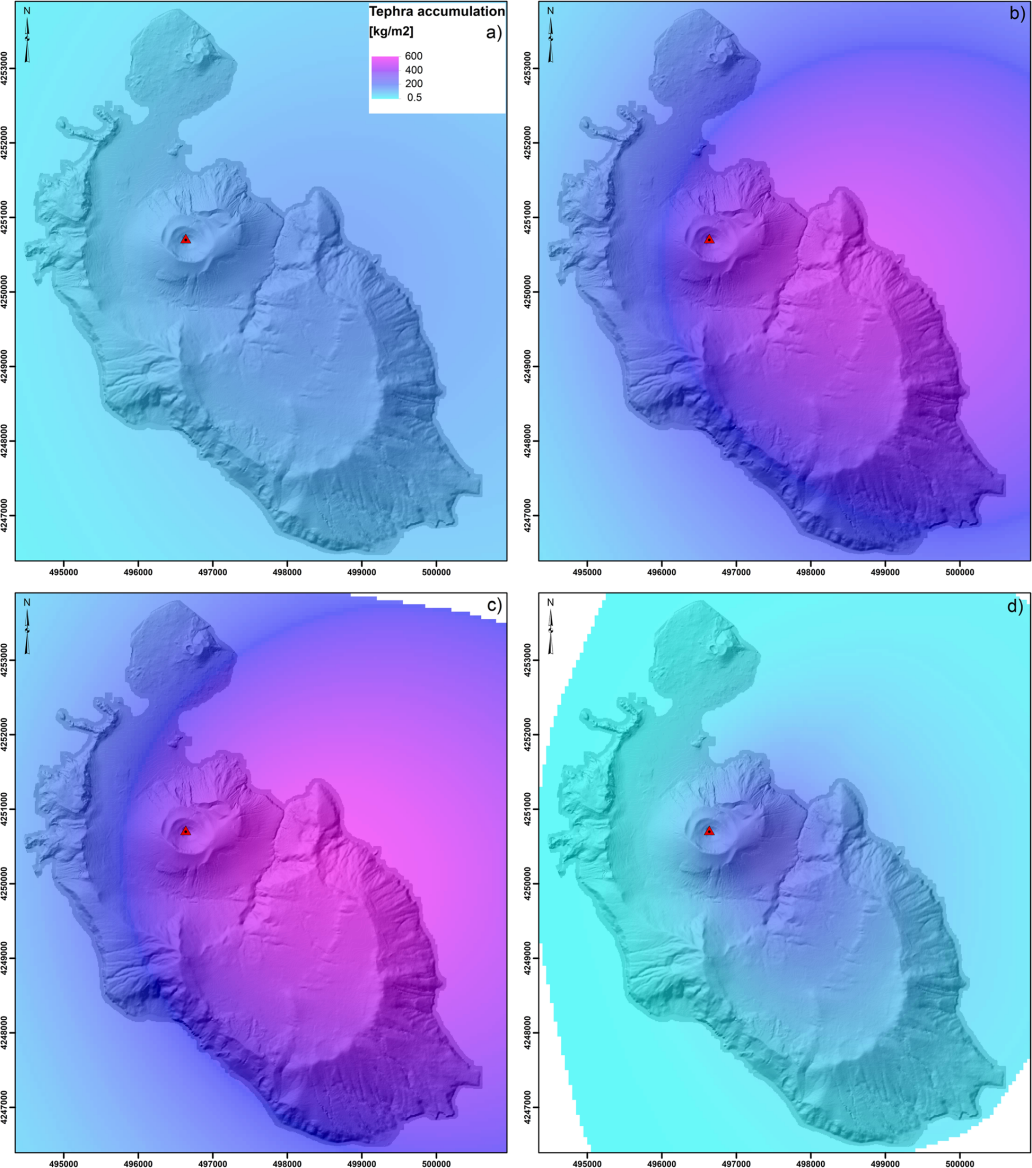
Probability isomass map of tephra fallout compiled for 25% of occurrence of a Vulcanian cycle (duration between 1 and 36 months) showing the accumulation after a) 6 months (real range 10.9 to 158.2 kg/m^2^; b) 24 months (real range 35.9 to 524,8 kg/m^2^; c) and 36 months (real range 50 to 600 kg/m^2^; and d) probability isomass map of tephra fallout compiled for 25% of occurrence of a VEI 2 scenario (real range 0.5 to 250 kg/m^2^). Values are between the minimum and maximum values found for the 4 scenarios, i.e. 0.5 to 600 kg/m^2^

The hazard related to tephra fallout was assessed using a scenario-based approach and applying the probabilistic eruption scenarios described by Biass et al. (2016a) (Fig. 3). This means that the assessment is based on an assumption that the selected scenarios are going to happen (i.e. conditional probability). In particular, we focus on the tephra fallout associated with two main eruptive styles of the past 1000 years: a long-lasting Vulcanian eruption such as that of 1888–90 and a subplinian eruption, which emplaced deposits such as the Palizzi D sub-unit (Volcanic Explosivity Index, VEI, 2) (e.g., Di Traglia et al. 2013; Biass et al. 2016a). The modelling was performed using the advection-diffusion model TEPHRA2 in the TephraProb framework (see Biass et al. 2016c for details on the hazard assessment of tephra fallout). The Vulcanian cycle scenario is more complex than the subplinian scenario because similar or even higher accumulations can be reached but over a substantially longer time (i.e. 2–3 years for a Vulcanian cycle in contrast to a few hours for a VEI 2 subplinian eruption; Biass et al. 2016a). As a result, the long-term management of a Vulcanian cycle is complicated by the long duration of activity that impacts the system with interaction between primary (tephra fallout) and secondary (lahars) hazards (Baumann et al. 2019). Hazard scenarios were modelled as Eruption Range Scenario (ERS) that is, all eruptive and atmospheric parameters are determined stochastically (Biass et al. 2016a). Eruption source parameters of the Vulcanian ERS are similar to the 1888–90 eruption with eruptive cycles lasting between 1 and 36 months. The ERS VEI 2 subplinian eruption was modelled with eruption source parameters derived from the Pal B and Pal D eruptions (Di Traglia et al. 2013). The ADVISE model requires hazard data to quantify hazard intensity metrics. For the tephra hazard, we use probabilistic isomass maps from Biass et al. (2016a), which quantify the tephra accumulation that occurs at a given percentile over all simulations of the probabilistic ERS scenario. In particular, we here use an arbitrary probability threshold of 25%, implying that only 25% of the total simulations produced larger accummulations; however, the method can be applicable with any probability threshold of scenario-based hazard assessments or long-term probabilistic hazard analyses. Figure 3a, b and c show the evolution of tephra-fallout accumulation for the Vulcanian scenario 6, 24 and 36 months after eruption onset.

Due to the prevailing W and NW winds, all maps show a dispersal towards E-SE and the accumulation associated with the Vulcanian scenario after 24 and 36 months are significantly higher with respect to that of a short-lived VEI 2 subplinian eruption. We selected the Vulcanian scenario with accumulation shown after 6 months and the VEI 2 subplinian scenario to discuss the risk assessment in an emergency management context. Similarly, we selected the Vulcanian scenario with accumulation shown after 36 months and the ERS VEI 2 subplinian scenario to discuss the risk assessment in terms of risk management. It is important to stress that, as an example, here we show only the maximum value of probability of dry tephra accumulation associated with long-lasting as we assume that no rainfall and clean-up occur between single Vulcanian explosions. This scenario is significantly different with respect to a situation where tephra accumulation on roof is removed between each explosion (e.g., Bonadonna et al. 2002). In addition, Biass et al. (2016) have shown how rainfall would increase the probability of reaching a certain mass/area by about 10–15%. However, for the sake of illustration of the model ADVISE and due to a lack of erosion model, here we prefer to focus on dry tephra accumulation with no-cleanup taking place between explosions.

### Elements at risk

Given the relatively small surface area of Vulcano, the whole island can be considered exposed to tephra fallout associated with an eruption of La Fossa volcano of the style described in the two previous sections (Fig. 3). Below we summarise the most relevant numbers related to the seasonally changing population and to the main assets on the island. Given that disaggregated data for Vulcano island are not available, the exposure analysis did not include the assessment of the economic value of the elements at risk.

#### Exposed population

##### Residents

Vulcano island is administratively dependent on the Municipality of Lipari. In 2017, its residential population reached 1282 people, with about 2% below 5 years of age, 4% between 5 and 10 years, and 26% above 65 years; 634 people reside in Porto, 389 in Piano, 212 in Vulcanello, 28 in Lentia and 19 in Gelso (Comune Lipari 2017). While 1282 represents the official population, the effective number is closer to 800, especially in wintertime, because all touristic activities cease and some 38% of the owners of hotels and residences return to winter homes either on Sicily or the European continent. During the middle and high touristic season (from April to October), between about 1000 and 28,000 tourists visit the island per month (Table 3), largely increasing the number of people present on the island. Italians mainly come in July–August, whereas foreigners and students come in March–June and September–October. Moreover, foreigners also come to work for hotels. This variability in origin of visitors should be considered when developing educational materials regarding volcanic risk and on emergency procedures. There is only one school on Vulcano. It receives children from primary to middle school (age of 14). Children then attend high school or university elsewhere (e.g. Lipari, Sicily or the mainland of Italy).

##### Visitors

Official statistics of both visitors and seasonal workers are not available for the island. Therefore, an estimate has been derived from data of passengers arriving to Vulcano by sea, either by ferries or ships, which represents the only possible means of transport. The only commercial port on the island is that in Porto Levante, which serves as the link between Vulcano and the rest of the archipelago and with selected harbours on the Italian peninsula (e.g. Reggio Calabria) and on Sicily (Milazzo and Messina). For the ordinary transfer of people and commercial goods there are several companies that provide daily service from early morning to late afternoon (mostly Siremar from Naples and Liberty Lines from Milazzo). The main boats used are hydrofoils (primarily for passengers) and larger shipping ferries for passengers, automobiles and most goods. Several small tourist companies also provide transport services and tours across the archipelago, including mini cruises around Vulcano Island (Table 4).

Passenger data to Vulcano island for the entire 2017 have been compiled, thanks to the collaboration of the main public/private transport agencies, and of the ports of the seven Aeolian islands that are connected to the mainland through the harbours of Milazzo, Reggio Calabria, Palermo, Messina and Naples (Table 4). The main tourism on Vulcano consists of daily tourism, with a large number of visitors that remain 1 day on the island as part of longer vacations spent on the other islands. Liberty Lines and Siremar companies transported a total of about 234,000 passengers to Vulcano in 2017, almost half of which was in the months of June, July and August. August certainly represents the most crowded month with a peak of about 77,000 arrivals counting data provided by all companies. On average, this constitutes arrival of about 2500 passengers on Vulcano daily in August (Table 4).

#### Built-up area

The Italian Census 2000 (ISTAT 2005) provides information related to buildings such as the number of floors, and the year of construction at the scale of aggregated areas for the five main inhabited areas (Vulcanello, Porto, Lentia, Piano and Gelso). Vulcano island presents a rather simple road network and few critical infrastructure and facilities: two power plants (one gasoline plant in Porto and one solar plant in Piano); a telecommunication system in Porto; a desalination plant and a waste water treatment plant in Porto; a medical ambulatory in Porto; a Carabinieri police station between Porto and Piano; a school in Piano; three churches (one each in Porto, Piano and Gelso); three ports and three heliports or helicopter pads, one in Vulcanello and Piano and one on La Fossa cone (Fig. 3). Airports and railway are absent, and the three heliports are only used for emergency or monitoring operations. The harbour system is, therefore, the key factor and a strategic infrastructure both for transportation to and from the island and to facilitate any emergency response. The three harbours on the island are Porto Levante, Porto Ponente and Porto Gelso. The main port is that of Porto Levante, while, due to shallow waters and poor maintenance, the other two are mostly used in case of emergency, although small boats dock at both. Selected strategic services are also based on ferryboats such as the transport of vehicles for collecting solid waste, the transport of the fuel supply from Milazzo, and the fireman brigade from Lipari. A relatively new desalination plant is not presently in function, so water supply to the island is made through tankships, which moors offshore near Porto Levante and pipes water onto the island.

### Vulnerability

In order to illustrate the methodology, we focus our analysis on residential buildings and on the critical infrastructure permitting accessibility within and to the Island (i.e. the transport system: roads, ports and heliports).

#### Physical vulnerability

##### Residential buildings

A dedicated matrix was adopted comprising 20 parameters related to: a) type of use (residential, commercial, public facilities, etc.); b) number of floors; c) roof features (flat or pitched); d) roof span; e) construction technique (masonry, reinforced concrete, mixed); f) building morphology (regular and irregular); and g) its maintenance (poor, good). For each hazard, the relevance of each parameter is assessed separately and attributed a score ranging from 0 to 1. For example, in the case of tephra fallout, the most critical element is the roof characteristics (e.g. material, angle, span and maintenance), which together determine the capacity of the roof to sustain loading by tephra. Based on the 1996 topographic map, Vulcano has 1093 buildings, most of which were built between 1972 and 1981 (ISTAT 2005). The typology of existing buildings was estimated in 2011 through the field survey of the most representative building within a 100 × 100 m grid. Some 255 buildings were assessed, representing 23% of the 1996 building stock. Most of the surveyed buildings are residential houses, occupied either on a yearly basis or more often during the summer season (May–October). Most buildings (73%) have flat roofs and 70% are composed of only one floor. It is very difficult to assess construction materials based on visual inspection only, so only 12% were completed. The vulnerability of roofs to tephra fallout was therefore assessed using the work of Spence et al. (2005), which derives vulnerability curves for European buildings. Based on the building sampling and preliminary analytical work on roof behaviour of Vulcano (Chevalley and Hänggeli 2011; Liu 2011; Boillet et al. 2012), we assumed that buildings have either flat reinforced concrete roofs or tiled roofs over a timber structure in different conditions (poor, average or good). The 4 main roof types identified and the corresponding mean values necessary for roof collapse (tephra load) are: tiled roof with poor condition (Weak (WE); 2.0 kPa); tiled roof with average or good condition (Medium weak (MW); 3.0 kPa); flat reinforced-concrete roof with average condition (Medium strong (MS); 4.5 kPa); flat RC roof, good condition (Strong (ST); 7.0 kPa) (Biass et al. 2016a).

##### Transport system

**Fig. 4:**
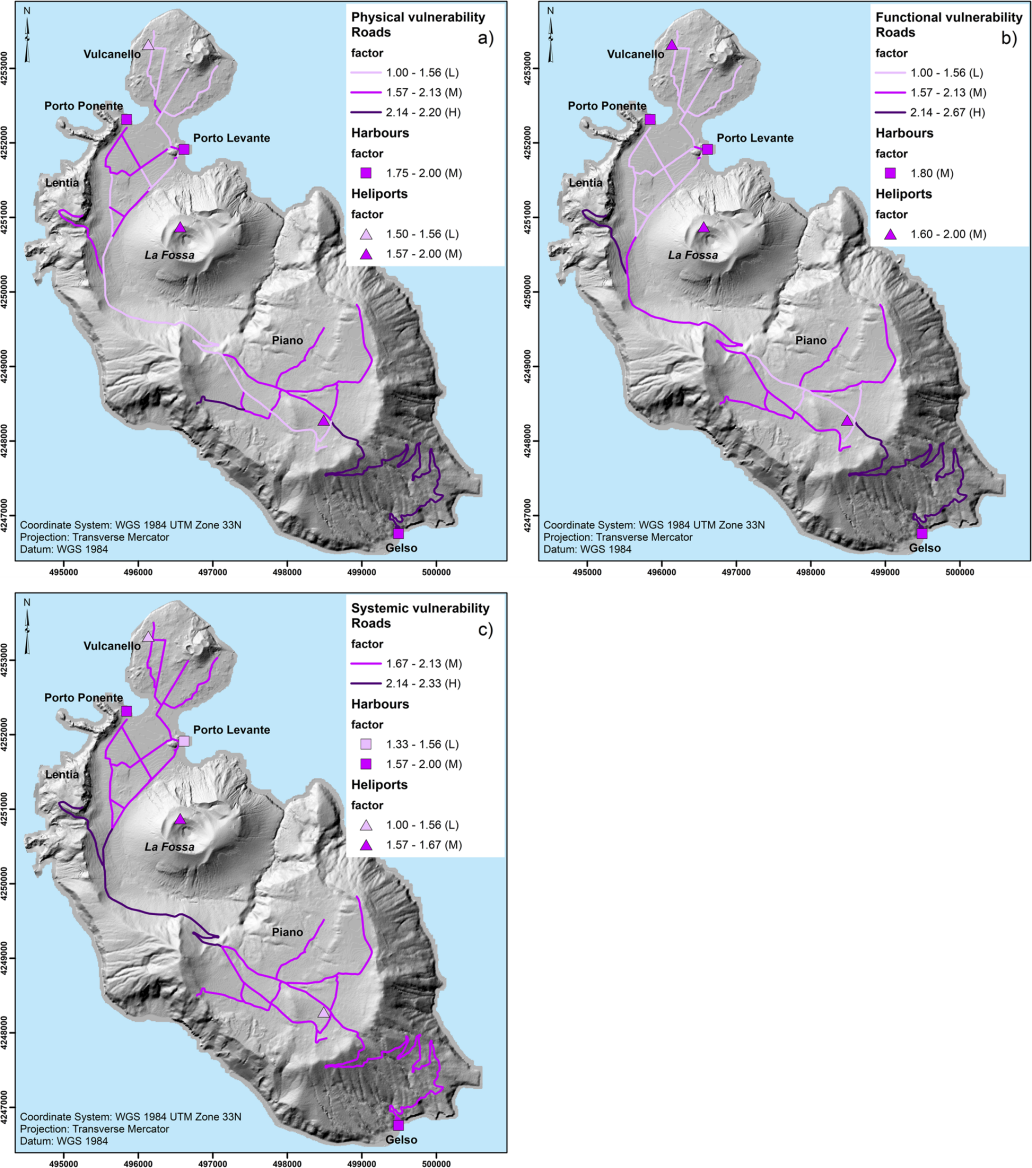
**a** Physical vulnerability, **b** functional vulnerability, and **c** systemic vulnerability of the transport system. The assessment is based on the qualitative levels (L, M, H) of Tables 5, 6 and 7. Nonetheless, each qualitative level is assigned a value (1, 2, 3; Tables 5, 6 and 7) in order to obtain a quantitative classification (indicated with the three different colours from rose to dark violet). Values associated with the three categories are also indicated in legend

In addition to intrinsic structural elements such as material of construction, the physical vulnerability of infrastructure also considers external factors that could influence their resistance (e.g. level of protection). A general example of the matrix used to assess individual aspects of roads, harbours and heliports is shown in Table 5. The majority of the roads are paved with asphalt, however, in some remote areas, roads are only graded. These are normally used to reach livestock and vineyards. Most roads lack drainage grates to convey surface water runoff, which increases the probability of flood during intense rainfall events. The only drainage grates are located along the Provincial Street 179 that passes at the bottom of La Fossa cone, where loose ash and lapilli sediment out from erosion of the north flank of La Fossa cone occurs. The complete assessment of physical vulnerability is shown in Fig. 4a, where the areas with highest vulnerability for roads are those reaching Porto Gelso and Monte Saraceno. All of the ports display a medium vulnerability (values derived from Table 5 and used to compile Fig. 4a are available in supplementary material). In particular, the road to Porto Gelso is affected in some parts by local landslides and severe cracks on the pavement. For comparison purposes, low, medium and high vulnerability levels displayed in Fig. 4 are assigned by dividing the maximum and minimum values of the average obtained across the three vulnerabilities’ matrices in three categories (see Table S.1, S.2 and S.3 of Supplementary Material). Individual values are kept for the combinaison with hazard categories to obtain the specific damage (physical, functional and systemic) (see following sections).

**Table 5:** Matrix used to assess the physical vulnerability of roads, harbours and heliports on Vulcano (adapted from Guobadia 2017). L, M and H indicate low, medium and high (Qualitative assessment). Numbers used for a quantitative vulnerability assessment are also indicated in the fifth column

System	Aspect parameters	Criteria for assessment	Physical vulnerability indicator	Physical vulnerability level and associated score
**ROADS**	Interaction with vulnerable buildings or areas	Close or far from vulnerable buildings/ close or far from vulnerable areas	No interaction (L) Partial interaction (M) High interaction (H)	1 (L), 2(M), 3(H)
	Level of protection	In a protected or not protected place	Protected (L), Average protection (M) Non protected (H)	1(L), 2(M), 3(H)
	Maintenance	Evaluation of the level of maintenance, visual survey, maintenance document	Good maintenance (L) Low maintenance (M) No maintenance (H)	1(L), 2(M), 3(H)
	Water drainage	Presence of a grid, connections, sewers	Existing (L) Partial (M) Non existing (H)	1(L), 2(M), 3(H)
	Pavement/Construction assessment	Material/production methods	Good (L) Average (M) Not good (H)	1(L), 2(M), 3(H)
**HARBOURS**	Interaction with vulnerable buildings	Close to / far from vulnerable buildings	Interaction (H), few Interaction (M) - No interaction (L)	1 (L), 2(M), 3(H)
	Level of protection	In a protected / not protected place	Protected (L), partially protected (M), not protected (H)	1(L), 2(M), 3(H)
	Maintenance	level of maintenance	Good (L), Low (M), No maintenance (H)	1(L), 2(M), 3(H)
	construction assessment	Type of structure, materials of construction	High (H), medium (M), low quality (L)	1(L), 2(M), 3(H)
**HELIPORTS**	Interaction with vulnerable buildings	Close to / far from vulnerable buildings	Interaction (H), few Interaction (M) - No interaction (L)	1 (L), 2(M), 3(H)
	Level of protection	In a protected / not protected place	Protected (L), partially protected (M), not protected (H)	1(L), 2(M), 3(H)
	Maintenance	level of maintenance	Good (L), Low (M), No maintenance (H)	1(L), 2(M), 3(H)
	Pavement / construction assessment	material	good (H), medium (M), poor (L)	1(L), 2(M), 3(H)

#### Functional vulnerability of the transport system

The transport system is recognized as a fundamental aspect of both risk and emergency management (e.g. Mossoux et al. 2019). The assessment of functional vulnerability focuses on aspect such as internal redundancy (at the level of the infrastructure), internal interdependency (at the level of the system) and on specific infrastructure features (e.g. curves, slope, adherance, width, visibility for roads and docking and landing facilities for harbours and heliports) (Table 6). Concerning roads, the Provincial Street 179 that links the Porto area to Piano is the only road between these two main areas and is characterised by two lanes with a couple of hairpin turns on the caldera escarpment. In contrast, in the areas of Vulcanello, Porto and Piano there is a good degree of road redundancy, and the majority of the streets have two lanes. In Porto centre, the largest tourist area, the passage is forbidden to vehicles. The only road leading to Gelso harbour has multiple sharp turns and as noted earlier, there are problems of stability due to landslides, especially after a fire that has occurred during the summer of 2015. The roadway in some parts is also overgrown with dense vegetation and this makes the path to reach the harbour difficult even in normal conditions. In addition, in the last hundred meters of the road, close to the Gelso harbour, the road width becomes smaller, and an emergency truck may have problems in manoeuvring; furthermore, next to the harbour there is parking only for a few light vehicles (cars). An investment to improve the functionality of the harbours in Porto (Levante and Ponente) was carried out by the Ministry of infrastructure and by the Italian Civil Protection Department on Vulcano in 2008 (Appendix A). In particular, the Porto Levante harbour was extended, while the Porto Ponente pier was renewed, in order to facilitate the evacuation of the population in the case of a volcanic crisis. Nonetheless, it is important to consider that the functionality of both harbours and heliports strongly depends on the weather, as they might become unusable in case of storms/rough sea. The functional vulnerability map highlights the critical roads of Gelso and Lentia, as well as the road that links the Porto area to Piano (Provincial Street 179) and display a uniform medium vulnerability for harbours and heliports (Fig. 4b) (values derived from Table 5 and used to compile Fig. 4b are available in supplementary material; Table S2).

**Table 6:** Matrix used to assess the functional vulnerability of roads, harbours and heliports on Vulcano (adapted from Guobadia 2017). L, M and H indicate low, medium and high (Qualitative assessment). Numbers used for a quantitative vulnerability assessment are also indicated in the 5th column

System	Aspect parameters	Criteria for assessment	Loss of function indicator	Loss of function level and associated score
**ROADS**	Internal redundancy (in the asset)	Number of structures or paths with the same function	Redundant (L) Partial redundancy (M) No alternative (H)	1(L), 2(M), 3(H)
	Internal interdependency (in the system)	Existence of items/assets that link functionality (geographical scale)	High (H) Medium (M) Low (L)	1(L), 2(M), 3(H)
	Curves and slope	Features of the road	Flat (L) steep (M) very steep (H)	1(L), 2(M), 3(H)
	Pavement	Quality of adherence	Good (L) Not good (M) Very bad (H)	1(L), 2(M), 3(H)
	Visibility	Distance of spatial view	Long (L) Average (M) Short (H)	1(L), 2(M), 3(H)
	Road width	Features of the road (e.g. for urban setting the width of one lane should be 2.75–3.25 m)	Large (L) Normal (M) Narrow (H) with respect to regulation	1(L), 2(M), 3(H)
**HARBOURS**	Internal redundancy (in the asset)	Number of structures or paths with the same function	Redundant (L) Partial redundancy (M) No alternative (H)	1(L), 2(M), 3(H)
	Internal interdependency (in the system)	Structures /components that link functionally to each other	High (H) Medium (M) Low (L)	1(L), 2(M), 3(H)
	Docking facility	weather dependent	Usable (L), partially usable (M), not usable (H) during storm	1(L), 2(M), 3(H)
		type of ships/boats	Any type (L), medium size (M), small boats (H)	1(L), 2(M), 3(H)
		distance of spatial view	High (L), medium (M), low (H) distance	1(L), 2(M), 3(H)
**HELIPORTS**	Internal redundancy (in the asset)	Number of structures or paths with the same function	Redundant (L) Partial redundancy (M) No alternative (H)	1(L), 2(M), 3(H)
	Internal interdependency (in the system)	Structures /components that link functionally to each other	High (H) Medium (M) Low (L)	1(L), 2(M), 3(H)
	Landing facility	weather dependent	Usable (L), partially usable (M), not usable (H) during storm	1(L), 2(M), 3(H)
		Type of helicopter	Any type (L), medium size (M), small helicopters (H)	1(L), 2(M), 3(H)
		Distance of spatial view	High (L), medium (M), low (H) distance	1(L), 2(M), 3(H)

#### Systemic vulnerability of the transport system

Critical aspects of systemic vulnerability include external interdependency (amongst lifelines), redundancy or accessibility and transferability (Table 7). For Vulcano island, interdependency has to be considered at two spatial scales. A scale internal to the transport system, i.e. between the main access to the island (heliports and ports) and the basic services that are located in the two main urban centres (Porto and Piano). The larger spatial scale considers the dependency of the island on the mainland (i.e. Sicily and Calabria regions for the Vulcano case) from where consumer goods and products arrive on ordinary basis (e.g. water supply) and, certainly, in case of emergency. Redundancy can be considered in relation to the possibility for the three components of the network system (i.e. ports, heliports, and roads) to provide a similar service of accessibility within and to the Island. Transferibility (i.e. the possibility to relocate one function or service) is less relevant for a small island such as Vulcano. In fact, from a functional point of view, any infrastructure could be relocated, for example, on neighbouring islands; however, such relocation represents a rather drastic solution that would imply the abandonment of Vulcano as a territorial entity.

**Table 7:** Matrix used to assess the systemic vulnerability of roads, harbours and heliports on Vulcano (adapted from Guobadia 2017). L, M and H indicate low, medium and high (Qualitative assessment). Numbers used for a quantitative vulnerability assessment are also indicated in the 5th column

System	Aspect parameters	Criteria for assessment	Systemic vulnerability indicator (among lifelines)	Systemic vulnerability level and associated score
**ROADS**	Transferability	Possibility that one function or service can be relocated in another place in case of need	High possibility (L) Medium possibility (M) Low possibility (H)	1(L), 2(M), 3(H)
	Redundancy	Existence of other assets with the same function	Availability of other assets (L) Partial availability (M) No availability of other assets (H)	1(L), 2(M), 3(H)
	External interdependency (among lifelines)	Existence of functionality links among lifelines	High (H) Medium (M) Low (L) intra-system interdependency	1(L), 2(M), 3(H)
**HARBOURS**	Transferability	Other assets with the same function	Good (L) Medium (M) No availability of other assets (H)	1(L), 2(M), 3(H)
	Accessibility	Possibility to use/access the asset	low (H) Medium (M) Good accessibility to the assets (L)	1(L), 2(M), 3(H)
	External interdependency	Different systems linked functionally to each other	low (L), medium (M), high dependency (H) on other assets	1(L), 2(M), 3(H)
**HELIPORTS**	Transferability	Other assets with the same function	Good (L) Medium (M) No availability of other assets (H)	1(L), 2(M), 3(H)
	Accessibility	Possibility to use/access the asset	Low (H) Medium (M) Good accessibility to the assets (L)	1(L), 2(M), 3(H)
	External interdependency	Different systems linked functionally to each other	low (L), medium (M), high dependency (H) on other assets	1(L), 2(M), 3(H)

One relevant feature of the road system on Vulcano is that there is only one road that links the Piano to the Porto area; there is also only one road that links Piano with Gelso harbour and one that links Porto with Vulcanello. Bridges, tunnels, and viaducts do not exist. The road network is strategic because it allows the rescue and evacuation of people from hazardous places and the total absence of redundancy may provoke the separation of the island in many isolated areas. However, roads’ function can be transferred, at least partially, to other means of transport, like boats and helicopters (redundancy). It is important to note that helicopters cannot be used during tephra fallout because of ash resuspension and potential damage to the engines and blades. As a consequence, the systemic vulnerability will still differ from one area to the others. Porto has several roads and two harbours (even though, due to shallow waters, Porto Ponente is used only for small tourist cruises and in case of emergency). Vulcanello and Piano have a heliport, whereas Gelso has a harbour. Lentia instead is linked with only one road to the main arterial road (the Provincial Street 179). As a result, the systemic vulnerability map for the transport system (Fig. 4c) shows the low redundancy and difficulty to connect Porto and Piano and to reach Lentia (values derived from Table 7 and used to compile Fig. 4c are available in supplementary material; Table S3). In Piano area as well as in Porto area, instead, there are more possibilities; the three harbours allow people to leave and reach the island and goods and vehicles to be transported. This is why the roads that connect Lentia to Porto and Porto to Piano display high systemic vulnerability, whereas the system of roads inside Porto, Piano and Vulcanello are characterised by medium vulnerability. Regarding heliports and harbours, they can function independently. However, their function can only be partially transferred to roads, in particular when it concerns the evacuation outside the island. Their accessibility is variable, and for these reasons the level of vulnerability varies (Fig. 4c).

#### Resilience of the transport system

For a transport system to be resilient in the face of a volcanic crisis, various actions must be taken (e.g., long-term structural mitigation measures, availability of resources for cleaning and repairing) to allow the system to remain functional during the volcanic crisis. However, as the last eruption occurred in 1888–90, well before the development of the current network, the controlling factors of resilience could not be assessed. Only some insights can be provided. For example, a structural mitigation measure was constructed along one of the main roads in Porto (just north of La Fossa cone) to drain excess of debris material in case of floods and lahars, which occur relatively frequently compared to eruption related hazards. Such a measure would certainly reduce the impact in case of tephra accumulation and subsequent rain. On the other side, nothing is currently implemented for repairing and/or cleaning after the occurrence of an eruption.

### Risk assessment

Depending on the objective and on the availability of data, a qualitative, a semi-quantitative or a quantitative approach approach might be preferred. It is important to stress here that all the risk analyses presented in this section can be considered as scenario-based risk assessments as they are based on scenario-based hazard maps (Biass et al. 2016a) and that all tephra accumulation values provided in the hazard maps are associated with a 25% probability of exceedance.

#### Qualitative risk assessment

**Fig. 5:**
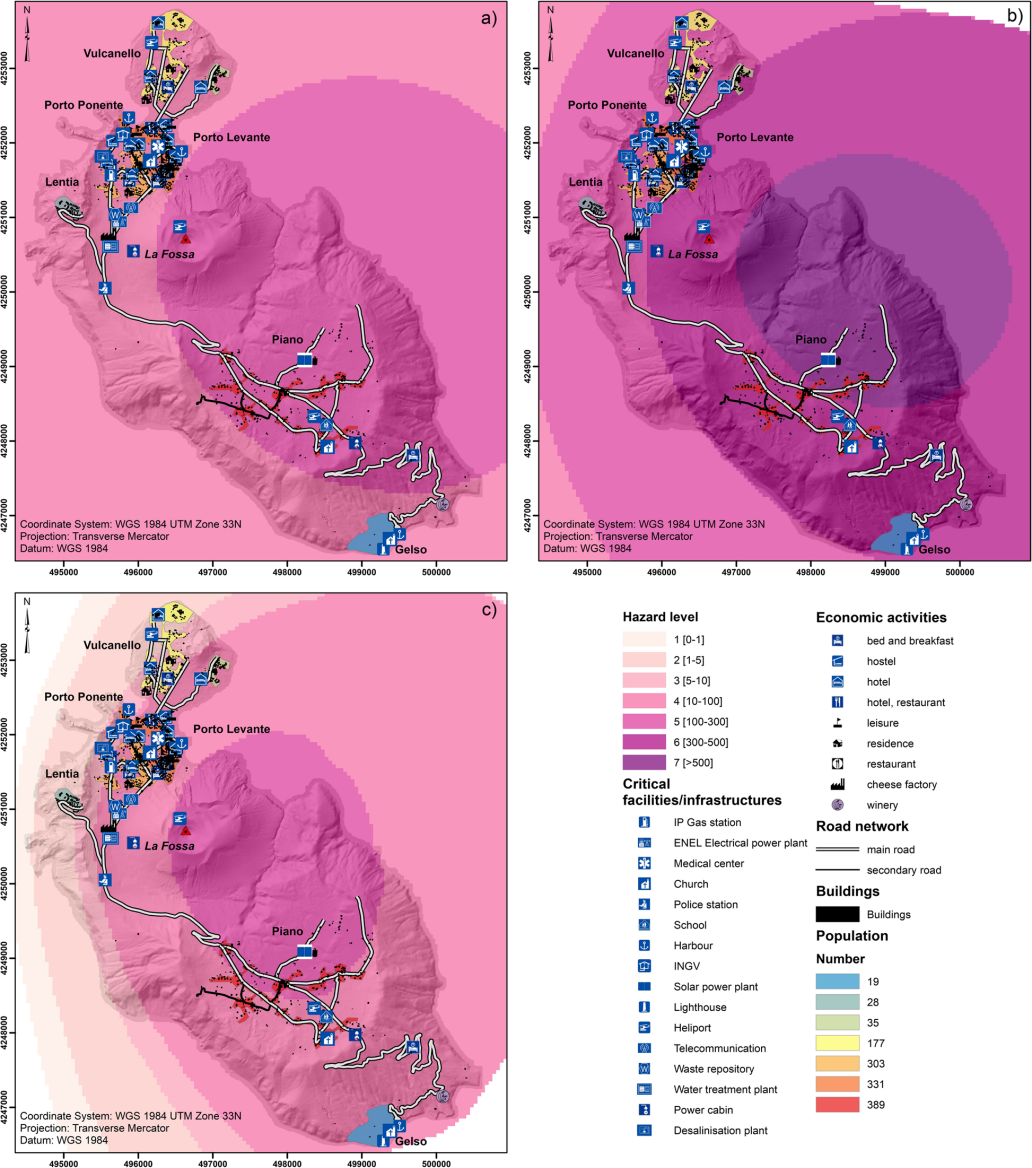
Exposure-based risk assessment based on the probabilistic isomass map for a 25% of occurrence of the 1–36 month Vulcanian scenario for a tephra-fallout accumulation after **a** 6 months (Fig. 3a) **b** and 36 months (Fig. 3c) and **c** of the VEI2 scenario (Fig. 3d) visualised for specific hazardous thresholds (see Table 8 for details in hazard thresholds)

**Fig. 6:**
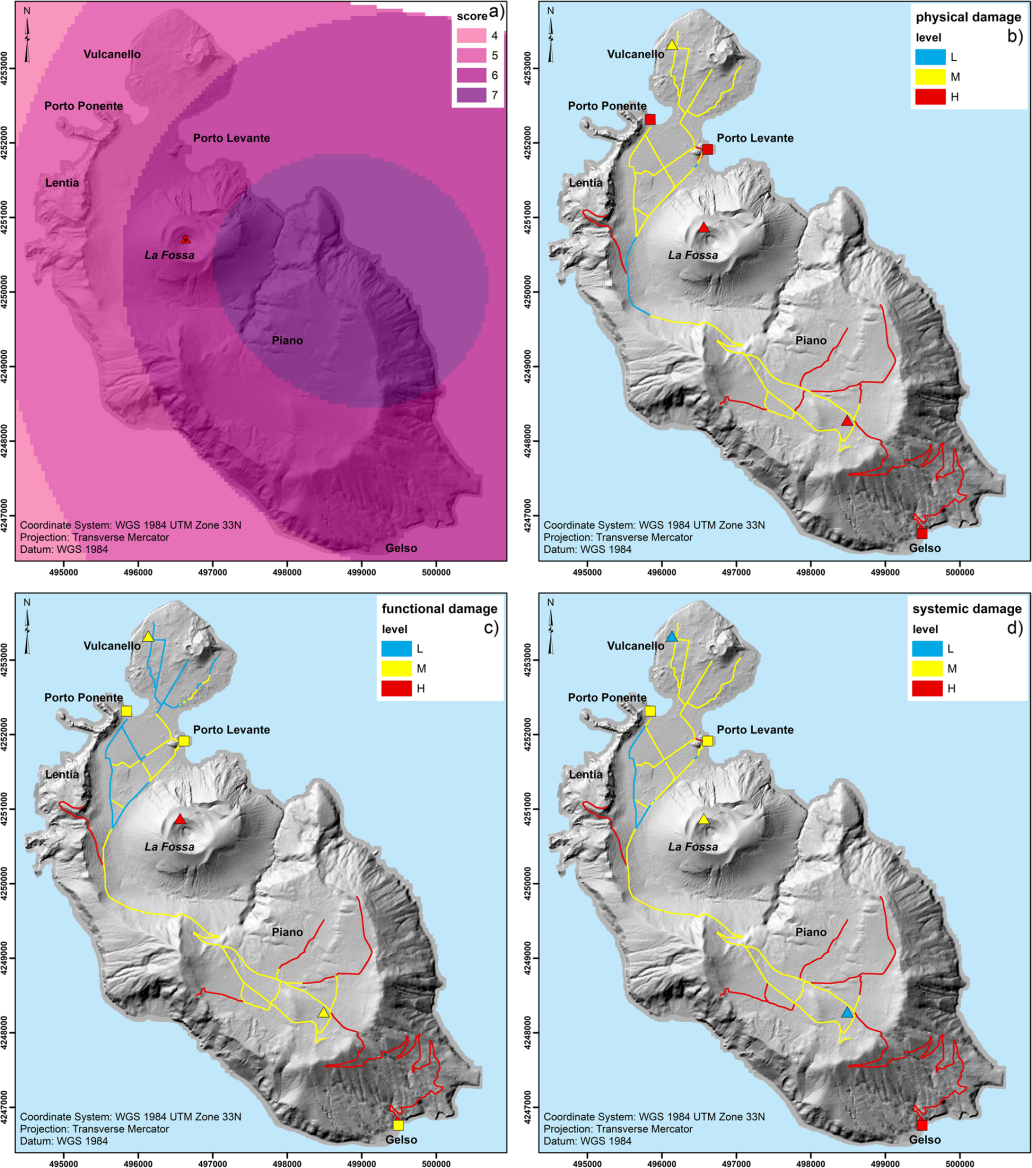
**a** Probabilistic isomass map for a 25% of occurrence of the 1–36 month Vulcanian scenario (accumulation after 36 months) visualised with hazard categories; **b** potential physical damage of roads, harbours and heliports obtained by combining Figs. 4a and 6a; **c** potential functional damage of roads, harbours and heliports obtained by combining Figs. 4b and 6a; **d** potential systemic damage obtained by combining Fig. 4c with Fig. 6a (see Appendix B for more details on the procedure to compile damage maps and Appendices C and D for the damage maps of the Vulcanian scenario after 6 months and the VEI 2 scenario)

Exposed elements could be simply overlapped with the probabilistic isomass maps of tephra accumulation to obtain an *exposure-based risk assessment* (Fig. 5). This *qualitative* approach does not require a detailed vulnerability assessment and is, therefore, relatively faster than a semi-quantitative and quantitative risk assessment (Fig. 1). Even though the information on local vulnerability is missing, general matrices that combine potential damage description with hazard thresholds (e.g. Table 8) can be used to provide some first-order insights into potential levels of severity of physical damage. Examples of information that can be extracted from an exposure-based risk map in the frame of emergency management are the location and numbers of critical infrastructure, such as roads, harbours and heliports, as well as the number of exposed people (Fig. 5; Table 9). Figure 6 can also provide information about the critical infrastructure that would be affected during an emergency. Given that during periods of volcanic unrest the style of the future eruption cannot be easily predicted, both the Vulcanian and VEI 2 scenarios are considered (Fig. 5a, c). Both maps show that the road to reach the Gelso harbour would be significantly affected by tephra deposits (accumulation from 10 to 300 kg/m^2^), which would certainly disrupt evacuation of people from Piano in case the evacuation is done during or after the eruption (Table 9). In fact, even though reduction of skid resistance in roads is maximized with tephra deposits between 1 and 5 mm thick (Blake et al. 2017a), visibility gradually decreases and wear of engine and abrasion of brakes and windscreen would gradually increase with increased tephra accumulation (Table 8). The heliport in Piano would also be covered by a thick tephra deposit (10 to 300 kg/m^2^, i.e. ⁓0.1 to 30 cm) that would make it either unusable or difficult for evacuation. This means that the people in Piano would be better evacuated from Porto di Levante in both a Vulcanian and a VEI 2 subplinian eruption. It is important to stress that in this case people would increase their exposure to proximal volcanic hazards, which could be especially dangerous in case of ballistic ejection and PDCs.

**Table 8:** Selected hazardous tephra-fallout accumulation thresholds (kg/m^2^) relevant for emergency and risk management on Vulcano in relation to buildings (Spence et al. 2005; Jenkins et al. 2014, 2015; Hayes et al. 2019), power systems/telecommunication (Jenkins et al. 2014, 2015) and roads/vehicles (Blake et al., 2017a,b; Jenkins et al. 2014; Wilson et al. 2017) and corresponding hazard score (1 to 7) used in Figs. 5 and 6. Tephra load (kg/m^2^) is converted in thickness assuming a deposit density of 1000 kg/m^3^. ND indicates no data available for this system. *Transport system mainly refers to Roads and vehicles, as no information is available for heliport and harbours; nonetheless, we can consider that disruption to airport occurs for thickness above 0.01 cm (Blake et al. 2017a)

**Buildings**	ND	No structural damage to buildings; possible infiltration and internal contamination and corrosion of metallic components; roofing materials may be abraded or damaged by human actions during ash removal		In rare instances, non-engineered and long span roofs may be vulnerable to damage, particularly when ash falls wet or is subsequently wetted; non-structural elements such as gutters and overhangs may suffer damage; some infiltration of dry ash into interiors	Structural damage; partial to complete collapse of weak (timber, corrugated metal) roofs	Structural damage; partial to complete collapse of concrete roofs	
**Power system / telecommunication**	ND	Temporary disruption of power system particularly with wet ash (e.g. flashovers); possible communication signal attenuation (e.g. radio); uninsulated lines may flashover.		Damage to telecommunication components and power cables through flashover; abrasion and or corrosion; failure of power generating plant (depending on system type and design); abrasion, clogging and flash-over causing disruption and/or damage to some electrical and mechanical equipment at substations	Damage to communication dishes and microwave towers due to excess of ash loading; structural damage to electrical distribution lines and support structures		Damage to communication dishes and microwave towers due to excess ash loading; permanent disruption and structural damage of power system
*Transport system	Minor skid resistance reduction possible and covering of markings	Skid resistance reduction likely and covering of markings; poor visibility; windscreen abrasion	Minor skid resistance reduction possible and covering of markings, poor visibility, windscreen abrasion	Minor skid resistance reduction possible and covering of markings; poor visibility; clogging of roadside drains and ditches; increased wear of engine and brakes and windscreen abrasion	Impassable for some vehicles and covering of markings; poor visibility. Dry, windy conditions exacerbate remobilisation and drifting.		
**Hazard score**	1	2	3	4	5	6	7
**Tephra load (kg/m**^**2**^**) / thickness (cm)**	0.1–1 (0.01–0.1)	1–5 (0.1–0.5)	5–10 (0.5–1)	10–100 (1–10)	100–300 (10–30)	300–500 (30–50)	> 500 (> 50)

**Table 9:** Exposed elements affected by tephra accumulation > 1 kg/m^2^ that can be derived from Fig. 5 (for all the scenarios and accumulation intervals) for emergency management purposes (1 kg/m^2^ is considered critical for evacuation operations; Table 8). Main roads harbours and heliports are from OpenStreetMap; number of residents is from Comune Lipari (2017), provided per area. It is assumed that the number is evenly distributed over the area; number of buildings is from Galderisi et al. (2013)

Exposed elements (tephra accumulation > 1 kg/m^**2**^)	Quantity
Main roads [km]	31.3
Harbours [Number]	3
Heliports [Number]	3
Residents [Number]	1282
Buildings [Number]	1093

On the contrary, the two harbours in Porto (Ponente and Levante) would be affected by a smaller tephra accumulation (5–100 kg/m^2^, i.e. ⁓0.5–10 cm). This would still affect evacuation operations, but it could be more easily managed. Finally, the heliport in Vulcanello would be affected by a tephra accumulation from 1 to 100 kg/m^2^, which may cause temporary closure (Table 8). It is important to notice that specific information related to damage associated with heliport and harbours are not available (Blake et al. 2017a). Potential damage to maritime infrastructure is better assessed based on sedimentation rate more than tephra accumulation, with 1 g/m^2^/h causing speed reductions and increased restrictions on vessel numbers in harbours and 500 g/m^2^/h causing most vessels to stop functioning due to impaired visibility (Blake et al. 2017a). Nonetheless, advection-diffusion models such as TEPHRA2 do not provide information on tephra sedimentation rate, and, therefore, only limited assessment can be made. Concerning heliports, we assume the same limitations apply as those for airports and road networks (e.g. Jenkins et al. 2015; Blake et al. 2017a, 2017b) (Table 8).

In the context of long-term risk management, we can go a step further by counting exposed elements as a function of specific hazard zones (Table 10), then prioritizing areas where mitigation measures could be implemented.

**Table 10:** Summary of the exposed elements associated with Fig. 5 (see Table 8 for references on hazardous thresholds). N indicates number; CIFs indicates Critical Infrastructure and Facilities; the symbol (*) indicates when certain levels of hazards are exceeded for individual scenarios (as tephra-fallout accumulation is larger than corresponding thresholds); the symbol (−) indicates when certain levels of hazards are not present

Scenario	Element at risk	Tephra hazard zones: hazard score / accumulation threshold [in kg/m^**2**^]						
		1 [0.1–1]	2 [1–5]	3 [5–10]	4 [10–100]	5 [100–300]	6 [300–500]	7 [> 500]
**Vulcanian** **6-month accumulation**	**Roads [km]**		14.9*		14.9	16.4	–	–
	**Buildings [N]**		749*		749	344	–	–
	**CIFs [N]**		15*		15	5	–	–
**Vulcanian** **36-month accumulation**	**Roads [km]**			9.8*		9.8	18.3	3.2
	**Buildings [N]**			510*		510	501	82
	**CIFs [N]**			7*		7	12	1
**VEI 2**	**Roads [km]**	1.5*	1.5	2.2	22	5.6	–	–
	**Buildings [N]**	90*	90	155	712	136	–	–
	**CIFs [N]**	1*	1	3	14	2	–	–

For the Vulcanian scenario with accumulation after 36 months, most buildings in Piano would be affected by a tephra accumulation > 300 kg/m^2^ and, therefore, would be likely to experience collapse of most roofs. As mentioned earlier, these values of tephra accumulation do not consider clean-up operations. In fact, systematic clean-up operations would be clearly required to protect people and assets (e.g. Hayes et al. 2017). On the contrary, Vulcanello, Porto and Lentia would be affected by tephra accumulation between 100 and 300 kg/m^2^. As a result, roof collapse in these areas would probably only occur for weak roofs, but clean-up and/or protection measures would be required to guarantee that key critical infrastructure would keep functioning. Experimental studies have shown, for example, that even small tephra accumulations would result in damage to insulators and cables especially in case of wet ash (e.g. Wardman et al. 2012; Wilson et al. 2017). In case of a VEI 2 subplinian eruption (short-lived), the north side of Piano in the vicinity of the solar plant would be affected by accumulations between 100 and 300 kg/m^2^, indicating that collapses of the weakest roofs might occur. The southern side of Piano at the school and heliport and most of the Porto area north of the Piano would be affected by accumulations between 10 and 100 kg/m^2^, indicating that mitigation measures to avoid infiltration in buildings and protect and/or reinforce non-structural elements should be implemented as well as assuring the functioning of critical infrastructure and facilities.

#### Semi-quantitative risk assessment

Given the qualitative nature of some of the components creating the risk, such as vulnerability, a comprehensive risk assessment can often only be semi-quantitative (Fig. 1) and described as:

1 $$Risk=\sum_{h=1}^n\sum_{s=1}^m\left(\left({H}_h\times {PV}_s\right)+\left({H}_h\times {FV}_s\right)+\left(\left(\left({H}_h\times {PV}_s\right)+\left({H}_h\times {FV}_s\right)\right)\times {SV}_s\right)\right)$$

where *h* represents a given hazardous phenomenon (e.g. PDCs, lava flows, tephra fallout) and *s* a given exposed system. *H*_*h*_ indicates the intensity of a given hazard *h*, *PV*_*s*_, *FV*_*s*_ and *SV*_*s*_ indicate the physical, functional and systemic vulnerability of a given exposed system, respectivelyas indicated in Fig. 1. Notice that each vulnerability dimension for each exposed system (*PV*_*s*_, *FV*_*s*_ and *SV*_*s*_) is determined as a mean value based on the number of associated indicators of each system (*N*_*PVs*_, *N*_*FVs*_ or *N*_*SVs*_) and indicator score *w*_*i*_; in particular, ${PV}_s=\frac{\sum_{i=1}^{N_{PV s}}\left({w}_i\right)}{N_{PV s}}$, ${FV}_s=\frac{\sum_{i=1}^{N_{FV s}}\left({w}_i\right)}{N_{FV s}}$, ${SV}_s=\frac{\sum_{i=1}^{N_{SV s}}\left({w}_i\right)}{N_{SV s}}$ (e.g. Tables 5, 6 and 7 and Tables S1, S2 and S3 in Supplementary Material). Additionally, (*H*_*h*_ × *PV*_*s*_) represents the potential physical damage, (*H*_*h*_ × *FV*_*s*_) represents the potential functional damage and (((*H*_*h*_ × *PV*_*s*_) + (*H*_*h*_ × *FV*_*s*_)) × *SV*_*s*_) represents the potential systemic damage. In this case, the various damages are summed instead of multiplied so that in case one of them is zero the risk is associated with those that are not zero. Note also that the systemic damage does not directly depend on the hazard alone (as for the physical and the functional damage) but on the physical and the functional damage. A comprehensive risk analysis should also include resilience (Fig. 1). However, as already mentioned, resilience of the transport system on Vulcano could not be quantified, and, therefore, was not considered in the semi-quantitave risk assessment (i.e., Eq. 1 and Fig. 8).

The first step involves analysis of our single hazard (tephra fallout) with the physical vulnerability of our single exposed infrastructure (transport system) (Fig. 4a). To facilitate the calculation, we converted the scenario-based hazard maps of Fig. 3 into scenario-based hazard maps with seven hazard categories applying a score from 1 to 7 based on Table 8 (Fig. 6a shows the example of the Vulcanian scenario). The potential physical damage map (Fig. 6b) is obtained by combining Figs. 4a with 6a (see Appendix B for the details of the procedure to compile the potential damage maps of Fig. 6). We consider that even a small accumulation of tephra (i.e. > 10 kg/m^2^) on roads can clog the water drainage and damage the lifeline (Table 6). The whole island is affected by this tephra accumulation in the Vulcanian scenario; therefore, the physical damage pattern for roads (Fig. 6b) is mainly controlled by the intensity of the hazard (see also Appendices C and D). Roads that result in the highest physical damage are mainly located in the Piano and Gelso areas due to the prevailing wind direction to SE.

The potential functional damage map (Fig. 6c; see also Appendices C and D) was compiled combining the hazard map (Fig. 6a; see also Appendices C and D) with the functional vulnerability map (Fig. 4b). For example, visibility on the roads might be reduced in case of tephra fallout, particularly in those parts where visibility is already poor due to the characteristics and state of the path. Tephra fallout may severely disrupt the transport system over large areas for hours or days. Safe driving will become difficult or impossible as vehicle headlights and brake lights will be barely visible as well as safe docking and landing. There is little existing lighting of public spaces on the island. Pedestrian paths are lit with streetlights in the Porto centre and harbour areas are lit with several lamps. The deposit of fine ash on road surfaces may also reduce traction, particularly when the ash becomes wet. Ash deposits thicker than 1 mm will obscure or cover markings on roads that identify lanes, road shoulders, direction of travel, and instructions (for example, stop or slow) which may confuse and disorient drivers (Blake et al. 2016, 2017a, 2017b); this is even more true for tourists that are not familiar with the local road system (Table 6).

**Fig. 7:**
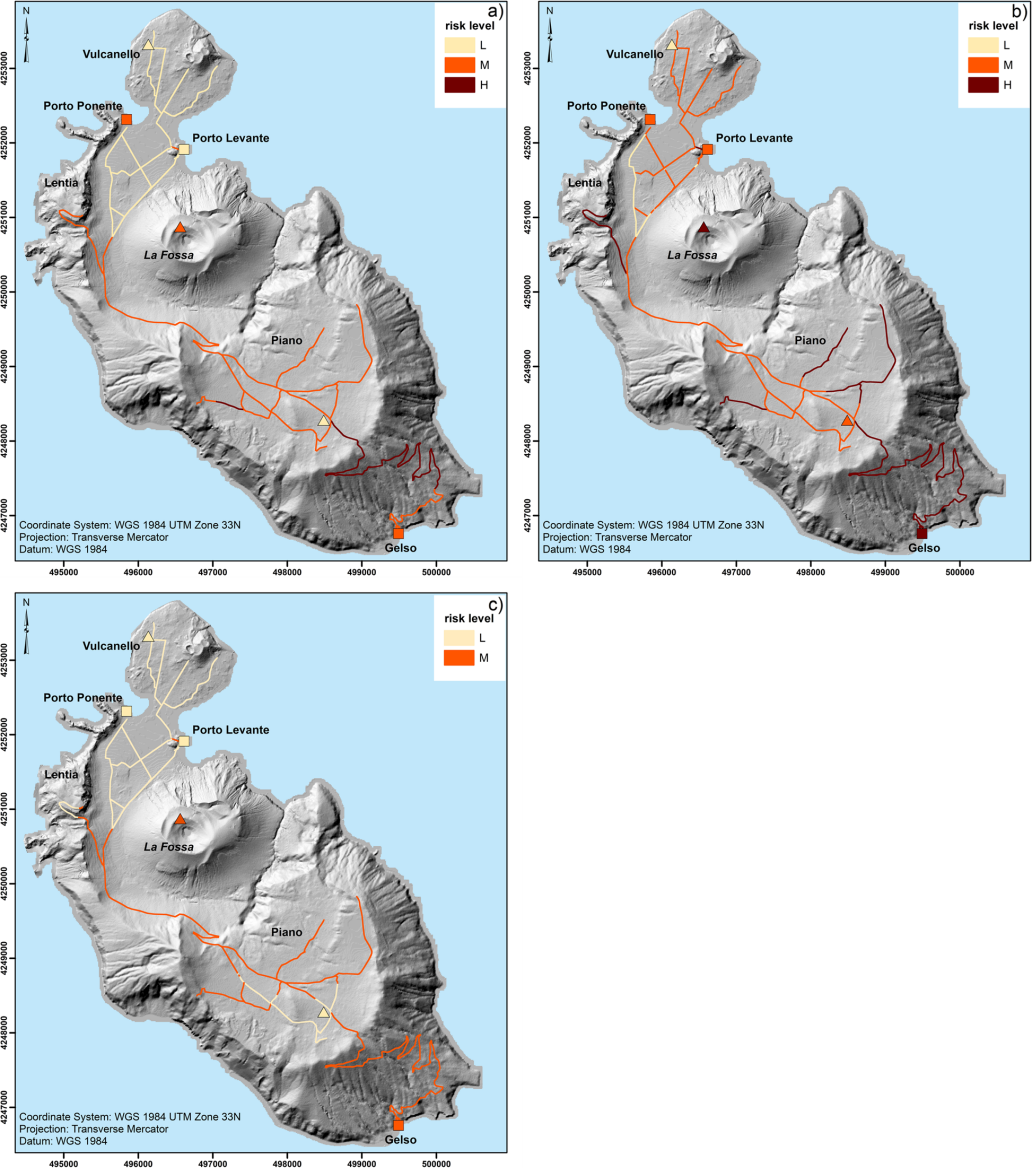
Risk assessment based on the ADVISE framework for the 1–36 month Vulcanian scenario after **a** 6 months, **b** and 36 months of accumulation, and **c** for the VEI2 scenario. The 3 classes are based on equal interval, using the minimum and maximum range of calculated values considering the 3 scenarios output ((xmax – xmin)/number of class)

**Fig. 8:**
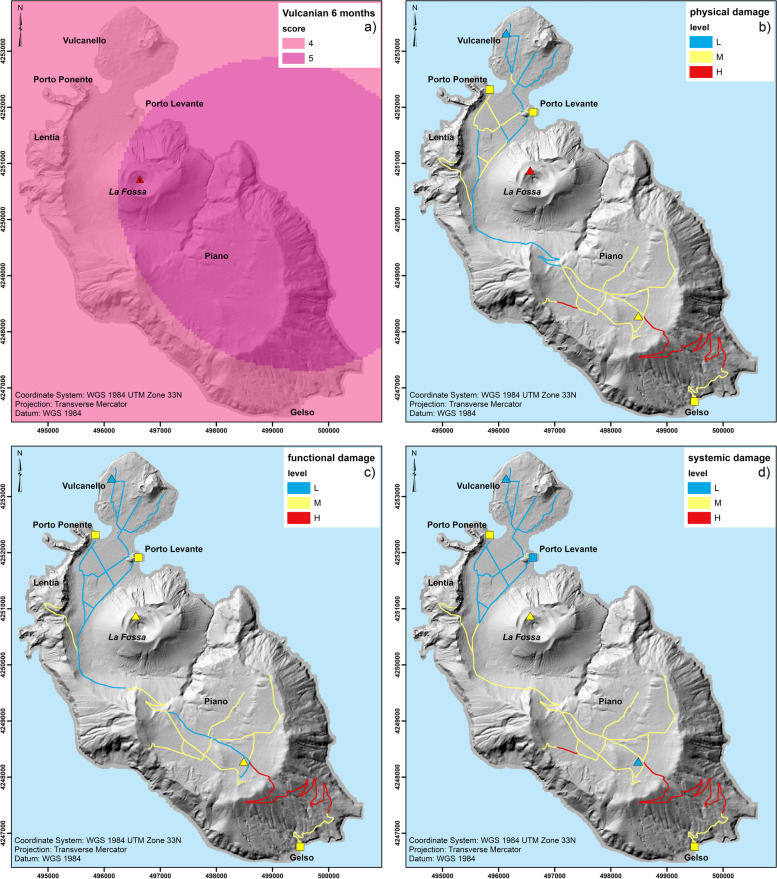
**a** Probabilistic isomass map for a 25% of occurrence of the Vulcanian scenario (showing tephra-fallout accumulation after 6 months) visualised with hazard categories; **b** physical damage of roads, harbours and heliports obtained by combining Figs. 4a and 8a; **c** functional damage of roads, harbours and heliports obtained by combining Figs. 4b and 8a; **d** systemic damage obtained by combining Figs. 4c and 8a (see Appendix B for more details on the procedure to compile damage maps)

The potential systemic damage (Fig. 6d) can be obtained by combining the hazard maps (Fig. 6a) with systemic vulnerability (Fig. 6c). The highest level of systemic damage is associated with the roads of Lentia and Gelso. In fact, both roads are characterised by high levels of functional vulnerability, due to the steep slopes, and narrow lanes that may become very slippery due to tephra fall. The road network in Vulcanello shows a medium systemic damage reducing accessibility to Porto di Levante (Fig. 6d). A comprehensive risk assessment requires the combination of physical damage with functional damage and systemic damage, as indicated in eq. 1 (i.e. by adding the values in Figs. 6b, c, d and 7).

This approach is also intrinsically *semi-quantitative* as qualitative assessment is combined with quantitative values (Tables 5, 6 and 7) to combine analyses such as hazard and vulnerability assessments. As quantitatively expressed in Eq. 1, such an assessment can be carried out for multiple hazards and multiple systems. In case of a semi-quantitative risk assessment, additional information can be derived both for emergency management and long-term risk management. As an example, for emergency management, such results permit identification of the weakest parts of the road system that could inhibit rescue/evacuation operations. This could include the road to Porto Gelso, which is essential during evacuation of the Piano area; Fig. 7). For long-term risk management, a semi-quantitative risk assessment provides information for parts of the road system that require intervention to maintain interconnection between infrastructure and between inhabited areas and critical infrastructure and facilities even during volcanic activity (e.g road network in Piano; Fig. 7).

#### Quantitative risk assessment

A *quantitative* risk assessment can also be carried out for specific elements (e.g. roofs). For Vulcano, we expressed the probability of roof collapse under a tephra load following the vulnerability framework proposed by the EXPLORIS project and field validation (Spence et al. 2005; Jenkins et al. 2014; Biass et al. 2016a). Although results show that a VEI 2 subplinian eruption has a virtually null probability of inducing roof collapse, the potential impact of a long-lasting Vulcanian eruption is significantly larger. When considering the deposit from an entire Vulcanian cycle (i.e. lasting up to 36 months and without cleaning; see Biass et al. 2016a for details), a median accumulation results in half of the building stock being exposed to a 20% probability of roof collapse. Without tephra-removal measures, the onset of collapse of the weakest roofs could occur 5–9 months after eruption onset. Clearly, this quantitative information related to the collapse of buildings is mostly relevant for long-term risk management strategies (e.g. land-use planning and structural mitigation measures to reinforce roofs in specific areas).

## Discussion

### Importance of an objective-based risk assessment

We have illustrated some of the complexities involved in analyses leading to risk assessments. In particular, a single strategy cannot be provided for all situations for two main reasons. First, the objective of risk assessments varies depending on whether they are for use in short-term emergency management or long-term risk management. Second, the availability of data varies. This latter point also relates to the urgency of the risk assessment and the time and resources available (i.e. both human and financial).

The objective of risk assessment is to provide information about the exposed elements and vulnerability dimensions of interest. Since both of these may vary in the case of short-term emergency management versus long-term risk management, the final risk analysis would be different for the two objectives (Tables 1 and 2). For example, in the case of the former, we would mostly consider those critical infrastructure and facilities required for evacuation (e.g. roads, heliports, and harbours in the case of Vulcano) as well as the people that will require evacuation (e.g. Table 9). The most important dimensions of vulnerability here are the functional and systemic vulnerabilities; in fact, we want to understand whether the key critical infrastructure and facilities required for evacuation can still function even in case of damage. In contrast to the emphasis on functional and systemic vulnerability in short-term emergency management, the most important dimension of vulnerability in long-term risk management is physical vulnerability, because it provides information about which buildings and critical infrastructure and facilities require strengthening in order to mitigate the stresses associated with specific hazards. However, systemic vulnerability is also important to assess in order to understand the interdependency, redundancy and transferability of infrastructure.

Given the variety of data available at any one time and the objectives, risk assessments can be qualitative, semi-quantitative or quantitative. Qualitative risk assessments such as exposure-based risk assessments (e.g., Fig. 5) provide insightful information, especially when few quantitative data are available (e.g. local data related to physical vulnerability). They may also become useful when a quick assessment is required, such as in the case of rapidly escalating volcanic activity, necessitating the compilation of an emergency plan. Qualitative risk assessment is also useful in instances where all available information is qualitative (e.g., systemic and social vulnerability). In some cases, qualitative information can be transformed into semi-quantitative or quantitative values (e.g., Tables 5, 6 and 7) to be combined with quantitative hazard assessment and produce a semi-quantitative risk assessment (e.g., Fig. 7). Finally, specific damage such as roof collapse can be analysed fully quantitatively in case a quantitative hazard assessment as well as fragility curves are available to link hazard and risk (e.g. Biass et al. 2016a).

In most cases, and as presented in this paper, a combination of qualitative, semi-quantitative and quantitative analyses are useful. In particular, the choice should be selected together with end-users such as political authorities, emergency managers and other stakeholders. This co-creation of a risk assessment is also important in order to engage the local community in the mitigation process as well as to help ensure that the most appropriate data available are utilized.

### Opportunities, limitations, and challenges of ADVISE model

The ADVISE approach includes consideration of elements for the compilation of risk assessment in the framework of both short-term emergency management and long-term risk management. It represents a useful tool for scientists, emergency managers and for those in charge of long-term risk management at the local level, especially in small touristic islands. Understanding the multi-layered vulnerabilities is essential when hazards are difficult to forecast well in advance of eruption, as in the case of many volcanic eruptions (Sarewitz et al. 2003). The advantage of the multiple steps that characterise the ADVISE approach is that emergency planners and/or land-use planners can better identify the reason(s) behind the high risk of a certain area, of a certain system or of a certain element of the system. In fact, often the final risk map does not provide information on which element to act to reduce risk (as information is not provided about what makes the risk high). Similarly, a risk map such as that of Fig. 7 only shows which elements of the road system are characterised by a high risk or which harbour or heliport can present a weak element of the transportation infrastructure. Nonetheless, all the information accumulated to compile the risk map of Fig. 7 (i.e. the vulnerability maps (Fig. 4) and the vulnerability matrices (Tables 5, 6 and 7) can be used to identify what factors affect the risk level and how the risk can be sustained or mitigated. For example, we can conclude that the Gelso road required for evacuation of the population of Piano during an emergency, is characterised by high risk because of high physical vulnerability (e.g. poor maintenance, lack of water drainage), high-medium functional vulnerability (e.g. small width, low visibility, many sharp turns, no internal redundancy) and high-medium systemic vulnerability (e.g. no redundancy within the whole infrastructure system). Such a road should be improved by acting on these specific elements. In case of Gelso port, risk reduction strategies should obviously also address the lack of a suitable staging area, which is currently very limited and could seriously impact evacuation operations for the Piano area.

The ADVISE model allows users to analyse long-term risk management strategies versus short-term emergency management strategies (Tables 1 and 2) in relation to a scenario-based hazard assessment. In particular, it is impossible to make accurate predictions of whether there will be an eruption and if so, what form it will take (e.g. Vulcanian or subplinian eruption). In case of long-term strategies, it is important to assess the actual probability of the scenario against its expected severity and decision makers can decide whether to give priority to the worst-case scenario or the most probable scenario. In case of short-term emergency management either a cumulative risk assessment that includes all possible scenarios or a series of individual risk assessments for each possible scenario should be considered (depending on the request of the local civil protection) unless the unrest phase has provided insights into the most likely scenario to happen. In addition, we cannot exclude that the unrest phase can produce physical, functional and systemic damage before the beginning of the climactic phase, which would worsen the operations for evacuation (e.g. soil deformation could make the Porto di Levante inaccessible to rescue ferries and boats). As a result, a hazard assessment associated with the unrest phase should also be carried out in order to produce a comprehensive risk assessment.

Regardless of the many benefits of the ADVISE approach, many aspects still require improvements. First, the strategy for the assessment of physical vulnerability needs to be better adapted to all systems (e.g. residential buildings, infrastructure, natural environment, social system, economic system). Second, evidence-based models are required for the assessment of systemic vulnerability for which knowledge and data are still insufficient. Third, a comprehensive strategy to better analyse and quantify resilience is necessary. Fourth, the integration between the physical description of damage and its translation into monetary terms is still very partial and would require collaborative work between engineers and economists. Finally, the ADVISE model can describe the impact of multiple hazards, and, therefore, also of cascading hazards such as tephra fallout and remobilisation of tephra deposit by water (i.e. lahars; e.g. Baumann et al. 2019). Nonetheless, as it stands, ADVISE does not account for the interaction between hazards.

Another important aspect to consider when assessing risk is scale. The ADVISE model is especially relevant at the local scale (as it has been shown for Vulcano island) but can also be applied at national scale. In fact, while the optimal application of ADVISE requires a large amount of data for the compilation of detailed hazard, exposure, vulnerability and resilience assessment, it can also be applied with incomplete datasets.

The lack of data is a general problem at multiple scales. In fact, while at global scales remote sensing data can provide important information but the detail of physical vulnerability cannot be resolved. At local scale many data are missing, as they are aggregated for a larger scale. This is the example of Vulcano that is part of the larger municipality of Lipari consisting of six islands (Alicudi, Filicudi, Lipari, Panarea, Stromboli, Vulcano); in addition, Lipari island is subdivided in five fractions (Canneto, Acquacalda, Quattropani, Pianoconte, Lami), meaning that Lipari Municipality consists of a total of 10 fractions. One of the main challenges in the application of the ADVISE model to Vulcano has been the difficulty in getting up-to-date and reliable data that are not aggregated for the whole municipality, including census data, economic data and information regarding the functioning of lifelines and infrastructure. This is not a condition that is specific to Vulcano; in fact, many islands in European countries would imply the same constraints. This limitation is worsened by the large fluctuation of population since tourism is often the main economic activity. However, an effective multi-hazard multi-system risk assessment can overcome the lack of data as it can be qualitative for certain systems and certain hazards and semi-quantitative or quantitative for others that are characterised by more information.

### Resilience considerations for Vulcano

Analyzing the resilience of different systems and at various scales can provide useful information for improving disaster risk management. Indeed, at the scale of the island, the resilience is mixed. On one side, social science interviews indicated that the main concerns of local populations are more related to the lack of cultural and social activities, provision of public services and strategic development strategy than to problems related to the volcano La Fossa (Galderisi et al. 2013). Moreover, hazard aspects have not been included in the development of the most recent Municipality Master Plan (2001), indicating either the lack of hazard awareness or the low priority given to hazard at the Municipality level. This was illustrated on Vulcano by the construction of the water treatment plant and the desalination plant in areas of high volcanic hazard. It is unclear to what extent these elements of the island’s infrastructure were considered in the various stages involved with their design, approval and execution. This real or apparent lack of consideration of volcanic hazards in the planning and approval process is a frequent occurrence for communities globally, despite international studies demonstrating that implemention of mitigation measures is a cost-effective strategy to help manage risk (e.g. UNISDR 2015; Wisner et al. 2003). On the other side, awareness and educational activities do exist (e.g. local volunteer associations such as vulcaniAMO: https://twitter.com/vulcaniamo, leaflets designed for tourists by the Italian Civil Protection (INGV-DPC 2013; http://www.ilvulcanoinforma.it/) and activities carried out with school children that involve local through national collaborations and representation: https://www.unige.ch/sciences/terre/fr/outreach/); in addition, the volcanic system on Vulcano island is monitored (e.g. Bonafede 1995; Gambino et al. 2007, 2012; Bonaccorso et al. 2010; Diliberto 2013; Ricci et al. 2015; Alparone et al. 2019) and various potential hazards are well studied and mapped, including PDCs, gas, tephra fallout, ballistic sedimentation, lahars (e.g. Ferrucci et al. 2005; Dellino et al. 2011; Baumann et al. 2019; Biass et al. 2016a, 2016b; Granieri et al. 2014). In such a context, the application of an integrated risk model such as ADVISE provides the opportunity to identify key mitigation measures to reduce volcanic risk and therefore increase resilience of the local community. By using several scenarios, interesting insights can be derived. For example, we found that long-lasting eruptions such as the Vulcanian scenario (i.e. with duration between 1 and 36 months) can generate more tephra accumulation than larger but shorter-duration eruptions (e.g. VEI 2 scenario). This clearly has implications in terms of long-term risk management, in particular, in relation to the necessity of implementing systematic clean-up operations for roads and roofs and waste management in order to avoid critical accumulations of tephra and pollution of the environment. Long-lasting eruptions are also associated with long-term health impacts such as anxiety and stress, and respiratory problems related to long-term exposure to volcanic ash (e.g., Jenkins et al. 2015). Structural mitigation measures identified based on the analysis presented in this work could include the reinforcement of building roofs in the Piano area as well as the improvement of road conditions and maintenance in this area to better secure people’s ability to navigate roadways during future crises. Protection from tephra accumulation and infiltration should also be considered for key infrastructure (e.g., the electrical power generation system and telecommunication) that could suffer significant functional damage even in case of small tephra accumulation.

## Conclusions

We have proposed a new model for the assessment of volcanic risk (ADVISE: integrAteD VolcanIc risk asSEssment) that addresses both emergency management and long-term risk management (Fig. 1). Such a model includes risk identification (i.e. hazard assessment, exposure assessment, vulnerability assessment and resilience assessment) as well as risk analysis (i.e. combination of various components of the risk identification). The exposed system includes natural environment, built environment, infrastructure, social system, and economic system. Risk analysis can produce a qualitative, semi-quantitative or quantitative risk assessment depending on the final objective and availability of data. In order to illustrate the various steps of the ADVISE model, we selected two eruptive scenarios (long-lasting Vulcanian and short-lived VEI 2 subplinian eruptions) for Vulcano Island (Italy) and tephra accumulation as one of the main associated hazard. The Vulcanian scenario has been explored at various tephra-fallout accumulation intervals (6, 24 and 36 months) to explore the evolution with time. In addition, we presented the exposure-based risk maps as an example of qualitative risk assessment, a risk assessment of the transport network system as an example of semi-quantitative risk assessment and the risk assessment of building roofs as an example of quantitative risk assessment.

Based on our analysis we can conclude that:

1. Risk assessments should be guided by specific objectives (emergency management and long-term risk management), by the time needed to compile the assessment and by the availability of data.
2. Different exposed elements and vulnerability dimensions might need to be considered depending on the objective of the risk assessment and the phase of the risk cycle.
3. Risk assessment should be co-designed with users in order to engage stakeholders and local community in the process of disaster risk reduction and increase resilience as well as to help ensure the use of correct and updated data in the analysis.
4. Risk assessment should be seen as a dynamic process as all factors (e.g. hazard, exposure, vulnerability, resilience) and systems evolve with time and at different speed.
5. The ADVISE model can be applied from local to national scale even though the type of data considered might differ.

### Supplementary Information

**Additional file 1.**

## Figures and Tables

**Fig. 9:**
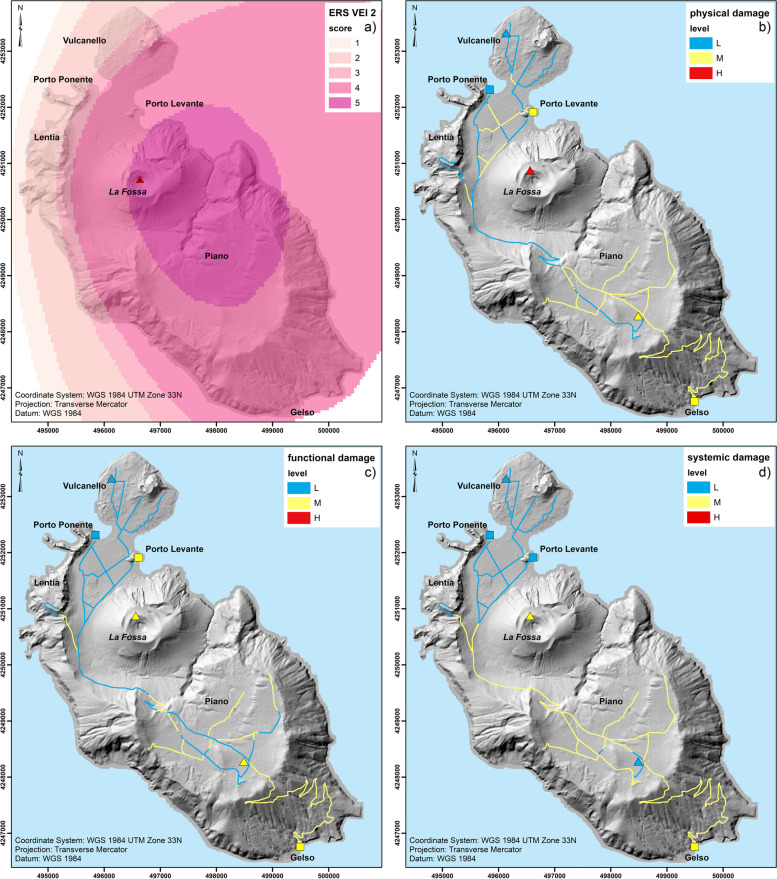
**a** Probabilistic isomass map for a 25% of occurrence of a VEI2 scenario visualised with hazard categories; **b** physical damage of roads, harbours and heliports obtained by combining Figs. 4a and 9a; **c** functional damage of roads, harbours and heliports obtained by combining Figs. 4b and 9a; **d** systemic damage obtained by combining Figs. 4c and 9a, b, and c (see Appendix B for more details on the procedure to compile damage maps)

## Data Availability

The datasets supporting the conclusions of this article are included within the article and its additional files.

## Appendix A

### Cost/benefit analysis of retrofitting Porto Levante and Ponente harbours

The financial resources of a community are limited. Decision-makers must thus make choices between different projects, including risk mitigation. Cost-benefit analysis (CBA) represents an important support tool for such decisions (e.g. Atkinson and Mourato 2008; Mechler 2016). The costs are given by the investment in risk mitigation and the operating costs such as the cost of building protections and their maintenance. The benefits are given by the “avoided costs” (e.g. the damage avoided thanks to the protections). Costs and benefits must be discounted as far as they appear in different periods of time, in order to obtain the project’s present cost. In principle, the *rate of discount* is given by a risk-free interest rate, adjusted by the risk brought about by the investment considered (e.g. Ramsey 1928). The second step is to consider the external costs and benefits (intangibles) in a qualitative or quantitative way. The third step is to make a trade-off between costs and benefits by taking into consideration the quantitative as well as the qualitative assessments. In making this trade-off, it has to be considered that the benefits, contrary to costs, are not immediately visible, i.e. they depend on the occurrence of the event.

An investment in risk mitigation was carried out by the Ministry of infrastructures and by the Italian Civil Protection Department on Vulcano in 2008. The Porto Levante harbour was extended, while the Porto Ponente pier was renewed, in order to facilitate the evacuation of the population in the case of a volcanic crisis. Other similar investments were carried out, for instance the improvement of the road that connects Porto Levante, Porto Ponente and Gelso. The cost (*C*) is assessed by means of the following formula:

$$C={I}_o+\sum_{t=1}^T\frac{c}{{\left(1+r\right)}^t}$$

where *I*_*o*_ represents the initial investment, c the annual maintenance costs, *r* the rate of discount and *T* the project’s lifetime.

The investment carried out in Vulcano amounted to € 1,327,465 as shown by official placards that were situated in front of the construction yard. In addition, we consider that, on average, the annual maintenance costs increase linearly from 0 to 1.5%, the installations’ lifespan is 20 years, and the social rate of discount is 3.75% (based on the estimate by Percoco (2008) for assessing public investments in Italy). If one reports the cost (*C*) to the population of Vulcano (1282 people), the annual expenditure related to this investment is € 82 per capita; if reported to the population of Lipari (12,815 people), the annual expenditure is € 8.2 per capita, while it is almost nil if reported to the population of Sicily. This shows the beneficial effect of sharing an investment in risk mitigation among a large population.

The expected benefits of the harbour extension and pier renewal are given by the safe evacuation of the population. To the extent that it is possible to quantify the number of casualties and injuries and the value of human life (Cropper and Sahin 2009), it would be also possible to monetize the benefits (the avoided costs) of the evacuation. This might help to make a trade-off between costs and expected benefits. Nonetheless, it must also be pointed out that such an investment has also improved the access to the island for both residents and tourists in regular times, representing a successful example of combination between risk management and socio-economic development.

## Appendix B

### Determination of physical, functional and systemic damage

All criteria used for assessing the different dimensions of vulnerability of the transport networks (physical, functional, systemic) have been scored from 1 to 3, indicating an increase in vulnerability (Tables 5, 6 and 7). Then for each component of the transport networks, the values have been added and then divided by the number of criteria to obtain the mean value (Supplementary Material Tables S1, S2, S3). To obtain the damage maps, vulnerability and hazard values have been combined using the Eq. 1. In order to compare results from different scenarios, an equal interval classification scheme was used based on the following formula:

$$Equal\ interval\ value=\frac{V_{max}-{V}_{min}}{N}$$

with V_max_ = the highest value obtained, V_min_, the lowest value obtained and N = the number of classes, i.e. 3 in the present case. Therefore, physical damage, functional damage, systemic damage as well as total risk have an imposed range of values, combining the lowest and highest values across the 3 scenarios used (Table 11). Then, the qualitative term, low (L), medium (M) and high (H) is attributed to each class.

**Table 11:** Minimum and maximum value for physical, functional and systemic damage as well as for the total risk for Vulcanian (after both 6 and 36 months of tephra-fallout accumulation) and the VEI 2 scenarios

Scenario	Range	Physical Damage	Functional Damage	Systemic Damage	Total risk
Vulcanian (6-month accumulation interval)	Min	4	4	12.44	23.11
	Max	11	13.33	48.67	73
Vulcanian (36-month accumulation interval)	Min	6	5	15.5	31
	Max	13.2	16	58.4	87.6
VEI 2 subplinian	Min	2.8	2.33	6.2	12.4
	Max	10	10.67	40.44	58.4
*Equal interval value range*	*Vmin*	*2.8*	*2.33*	*6.2*	*12.4*
	*Vmax*	*13.2*	*16*	*58.40*	*87.6*

## Abbreviations

ADVISE: integrAteD VolcanIc risk asSEssment
CBA: Cost-Benefit Analysis
CERG-C: Specialisation Certificate for the assessment and management of Geological and Climate Related Risk; http://www.unige.ch/sciences/terre/CERG-C/
ERS: Eruption Range Scenario
*H*_*h*_: Intensity of a given hazard (Eq. 1)
*FV*_*s*_: Functional vulnerability of a given exposed system (Eq. 1)
*N*_*FVs*_: Number of indicators of functional vulnerability for a given exposed system (Eq. 1)
*N*_*PVs*_: Number of indicators of physical vulnerability for a given exposed system (Eq. 1)
*N*_*SVs*_: Number of indicators of systemic vulnerability for a given exposed system (Eq. 1)
*PV*_*s*_: Physical vulnerability of a given exposed system (Eq. 1)
*SV*_*s*_: Systemic vulnerability of a given exposed system (Eq. 1)
PDC: Pyroclastic Density Currents
UNDRO: United Nations Disaster Relief Organization
UNDRR: United Nations Office for Disaster Risk Reduction; https://www.undrr.org/
VEI: Volcanic Explosivity Index (Newhall and Self 1982)
*w*_*i*_: Score assigned to each indicator *i* (Eq. 1)
