# Supplementary material for: Integrating hazard, exposure, vulnerability and resilience for risk and emergency management in a volcanic context: the ADVISE model
Source: J Appl Volcanol. 2021 Nov 8;10(1):7. doi: 10.1186/s13617-021-00108-5 (PMC8596497; doi:10.1186/s13617-021-00108-5)
Supplement: Supplementary file 1 — Additional file 1. [file 13617_2021_108_MOESM1_ESM.docx]

**Supplementary Material (Tables used to assign values to physical, functional and systemic vulnerability)**

| ID | road | interaction | protection | Maintenance | w_drainage | t_construction | total | mean |
| --- | --- | --- | --- | --- | --- | --- | --- | --- |
| 0 | main road | 1 | 1 | 1 | 3 | 1 | 7 | 1.40 |
| 1 | main road | 1 | 1 | 1 | 3 | 1 | 7 | 1.40 |
| 2 | main road | 1 | 1 | 2 | 3 | 1 | 8 | 1.60 |
| 3 | main road | 1 | 1 | 1 | 1 | 1 | 5 | 1.00 |
| 4 | main road | 1 | 1 | 1 | 3 | 1 | 7 | 1.40 |
| 5 | main road | 1 | 1 | 1 | 3 | 1 | 7 | 1.40 |
| 6 | main road | 1 | 1 | 3 | 3 | 1 | 9 | 1.80 |
| 7 | secondary road | 1 | 1 | 2 | 3 | 1 | 8 | 1.60 |
| 8 | main road | 1 | 1 | 1 | 3 | 1 | 7 | 1.40 |
| 9 | main road | 1 | 1 | 1 | 3 | 1 | 7 | 1.40 |
| 10 | main road | 1 | 2 | 1 | 3 | 1 | 8 | 1.60 |
| 11 | main road | 1 | 2 | 1 | 3 | 1 | 8 | 1.60 |
| 12 | main road | 1 | 1 | 1 | 3 | 1 | 7 | 1.40 |
| 13 | main road | 1 | 2 | 1 | 1 | 1 | 6 | 1.20 |
| 14 | main road | 1 | 1 | 2 | 3 | 1 | 8 | 1.60 |
| 15 | main road | 3 | 2 | 3 | 2 | 1 | 11 | 2.20 |
| 16 | main road | 1 | 1 | 2 | 3 | 2 | 9 | 1.80 |
| 17 | secondary road | 1 | 1 | 2 | 3 | 1 | 8 | 1.60 |
| 18 | secondary road | 1 | 1 | 3 | 3 | 3 | 11 | 2.20 |
| 19 | main road | 1 | 1 | 2 | 3 | 2 | 9 | 1.80 |
| 20 | main road | 3 | 2 | 2 | 2 | 1 | 10 | 2.00 |
| 21 | main road | 1 | 1 | 2 | 3 | 1 | 8 | 1.60 |
| 22 | main road | 1 | 1 | 2 | 3 | 1 | 8 | 1.60 |
| 23 | secondary road | 1 | 1 | 2 | 3 | 2 | 9 | 1.80 |
| 24 | main road | 1 | 1 | 2 | 3 | 2 | 9 | 1.80 |
| 25 | main road | 1 | 1 | 2 | 3 | 1 | 8 | 1.60 |
| 26 | main road | 2 | 1 | 1 | 3 | 2 | 9 | 1.80 |
| 27 | main road | 1 | 1 | 1 | 2 | 1 | 6 | 1.20 |
| 28 | main road | 1 | 1 | 1 | 3 | 2 | 8 | 1.60 |
| 29 | main road | 1 | 1 | 2 | 3 | 1 | 8 | 1.60 |
| 30 | main road | 1 | 1 | 2 | 3 | 1 | 8 | 1.60 |
| 31 | main road | 1 | 1 | 2 | 3 | 1 | 8 | 1.60 |
| 32 | main road | 1 | 1 | 1 | 3 | 3 | 9 | 1.80 |
| 33 | main road | 1 | 1 | 1 | 3 | 1 | 7 | 1.40 |
| 34 | main road | 1 | 1 | 1 | 3 | 1 | 7 | 1.40 |
| 35 | main road | 1 | 1 | 1 | 3 | 3 | 9 | 1.80 |
| 36 | main road | 1 | 1 | 2 | 3 | 1 | 8 | 1.60 |
| 37 | main road | 1 | 1 | 1 | 3 | 1 | 7 | 1.40 |
| 38 | main road | 1 | 1 | 1 | 3 | 1 | 7 | 1.40 |
| 39 | main road | 1 | 1 | 1 | 3 | 1 | 7 | 1.40 |
| 40 | main road | 1 | 1 | 1 | 3 | 1 | 7 | 1.40 |
| 41 | main road | 1 | 1 | 1 | 3 | 1 | 7 | 1.40 |
| 42 | main road | 1 | 1 | 1 | 3 | 1 | 7 | 1.40 |

| OID | Heliport | interaction building | protection_level | maintenance | pavement | total | mean |
| --- | --- | --- | --- | --- | --- | --- | --- |
| 1 | Vulcanello_heliport | 1 | 3 | 1 | 1 | 6 | 1.5 |
| 4 | LaFossa_heliport | 1 | 3 | 2 | 2 | 8 | 2 |
| 5 | Piano_heliport | 1 | 3 | 1 | 2 | 7 | 1.75 |

| OID | Harbor | interaction building | protection_level | maintenance | construction | total | mean |
| --- | --- | --- | --- | --- | --- | --- | --- |
| 2 | PortoPonente_harbour | 1 | 3 | 3 | 1 | 8 | 2 |
| 3 | PortoLevante_harbour | 2 | 3 | 1 | 1 | 7 | 1.75 |
| 6 | Gelso_harbour | 2 | 3 | 1 | 1 | 7 | 1.75 |

**Table S1. Physical vulnerability**

| ID | road | i_redundancy | i_dependency | features_path | type_pavement | visibility | width | total | mean |
| --- | --- | --- | --- | --- | --- | --- | --- | --- | --- |
| 0 | main road | 1 | 1 | 1 | 2 | 1 | 1 | 7 | 1.17 |
| 1 | main road | 1 | 2 | 1 | 2 | 1 | 2 | 9 | 1.50 |
| 2 | main road | 1 | 1 | 1 | 1 | 1 | 1 | 6 | 1.00 |
| 3 | main road | 1 | 1 | 1 | 1 | 1 | 2 | 7 | 1.17 |
| 4 | main road | 1 | 1 | 1 | 2 | 1 | 1 | 7 | 1.17 |
| 5 | main road | 1 | 1 | 1 | 2 | 2 | 2 | 9 | 1.50 |
| 6 | main road | 1 | 1 | 1 | 3 | 1 | 2 | 9 | 1.50 |
| 7 | secondary road | 1 | 1 | 1 | 2 | 1 | 2 | 8 | 1.33 |
| 8 | main road | 2 | 2 | 1 | 1 | 1 | 1 | 8 | 1.33 |
| 9 | main road | 1 | 2 | 1 | 1 | 1 | 1 | 7 | 1.17 |
| 10 | main road | 1 | 1 | 1 | 1 | 1 | 1 | 6 | 1.00 |
| 11 | main road | 1 | 1 | 1 | 2 | 2 | 2 | 9 | 1.50 |
| 12 | main road | 1 | 1 | 1 | 2 | 1 | 2 | 8 | 1.33 |
| 13 | main road | 1 | 1 | 1 | 1 | 1 | 2 | 7 | 1.17 |
| 14 | main road | 1 | 2 | 1 | 2 | 1 | 2 | 9 | 1.50 |
| 15 | main road | 3 | 2 | 3 | 3 | 3 | 2 | 16 | 2.67 |
| 16 | main road | 1 | 2 | 1 | 2 | 1 | 3 | 10 | 1.67 |
| 17 | secondary road | 2 | 2 | 1 | 2 | 1 | 3 | 11 | 1.83 |
| 18 | secondary road | 3 | 2 | 1 | 3 | 1 | 2 | 12 | 2.00 |
| 19 | main road | 3 | 2 | 1 | 2 | 1 | 1 | 10 | 1.67 |
| 20 | main road | 3 | 2 | 2 | 2 | 3 | 2 | 14 | 2.33 |
| 21 | main road | 1 | 2 | 1 | 2 | 1 | 1 | 8 | 1.33 |
| 22 | main road | 1 | 1 | 1 | 2 | 2 | 2 | 9 | 1.50 |
| 23 | secondary road | 1 | 2 | 1 | 2 | 1 | 2 | 9 | 1.50 |
| 24 | main road | 3 | 2 | 1 | 2 | 1 | 3 | 12 | 2.00 |
| 25 | main road | 1 | 1 | 1 | 2 | 1 | 2 | 8 | 1.33 |
| 26 | main road | 1 | 1 | 2 | 3 | 2 | 2 | 11 | 1.83 |
| 27 | main road | 3 | 2 | 2 | 1 | 1 | 1 | 10 | 1.67 |
| 28 | main road | 1 | 2 | 1 | 2 | 1 | 1 | 8 | 1.33 |
| 29 | main road | 1 | 1 | 1 | 2 | 1 | 2 | 8 | 1.33 |
| 30 | main road | 1 | 1 | 1 | 2 | 1 | 2 | 8 | 1.33 |
| 31 | main road | 1 | 1 | 1 | 2 | 1 | 2 | 8 | 1.33 |
| 32 | main road | 1 | 1 | 1 | 3 | 2 | 2 | 10 | 1.67 |
| 33 | main road | 1 | 2 | 1 | 2 | 1 | 1 | 8 | 1.33 |
| 34 | main road | 1 | 1 | 1 | 2 | 1 | 1 | 7 | 1.17 |
| 35 | main road | 1 | 1 | 1 | 3 | 2 | 2 | 10 | 1.67 |
| 36 | main road | 1 | 1 | 1 | 2 | 1 | 2 | 8 | 1.33 |
| 37 | main road | 1 | 2 | 1 | 2 | 1 | 1 | 8 | 1.33 |
| 38 | main road | 1 | 2 | 1 | 2 | 1 | 1 | 8 | 1.33 |
| 39 | main road | 1 | 2 | 1 | 2 | 2 | 2 | 10 | 1.67 |
| 40 | main road | 1 | 1 | 1 | 2 | 1 | 2 | 8 | 1.33 |
| 41 | main road | 1 | 2 | 1 | 1 | 1 | 1 | 7 | 1.17 |
| 42 | main road | 2 | 2 | 1 | 1 | 1 | 1 | 8 | 1.33 |

| OID | Heliport | internal_redundancy | internal_interdependency | landing_weather | landing_type | landing_view | tot | factor |
| --- | --- | --- | --- | --- | --- | --- | --- | --- |
| 1 | Vulcanello_heliport | 2 | 1 | 3 | 1 | 1 | 8 | 1.6 |
| 4 | LaFossa_heliport | 2 | 1 | 3 | 3 | 1 | 10 | 2 |
| 5 | Piano_heliport | 2 | 1 | 3 | 2 | 1 | 9 | 1.8 |

| OID | Harbour | internal_redundancy | internal_interdependency | docking_weather | docking_type | docking_view | tot | factor |
| --- | --- | --- | --- | --- | --- | --- | --- | --- |
| 2 | PortoPonente_harbor | 2 | 1 | 3 | 2 | 1 | 9 | 1.8 |
| 3 | PortoLevante_harbour | 2 | 1 | 3 | 1 | 2 | 9 | 1.8 |
| 6 | Gelso_harbour | 2 | 1 | 3 | 1 | 2 | 9 | 1.8 |

**Table S2. Functional vulnerability**

| ID | road | transferability | redundancy | interdependency | total | mean |
| --- | --- | --- | --- | --- | --- | --- |
| 0 | main road | 3 | 1 | 1 | 5 | 1.67 |
| 1 | main road | 3 | 1 | 1 | 5 | 1.67 |
| 2 | main road | 3 | 1 | 1 | 5 | 1.67 |
| 3 | main road | 3 | 1 | 1 | 5 | 1.67 |
| 4 | main road | 3 | 1 | 1 | 5 | 1.67 |
| 5 | main road | 3 | 1 | 1 | 5 | 1.67 |
| 6 | main road | 3 | 1 | 1 | 5 | 1.67 |
| 7 | secondary road | 3 | 1 | 1 | 5 | 1.67 |
| 8 | main road | 3 | 2 | 1 | 6 | 2.00 |
| 9 | main road | 3 | 2 | 1 | 6 | 2.00 |
| 10 | main road | 3 | 1 | 1 | 5 | 1.67 |
| 11 | main road | 3 | 1 | 1 | 5 | 1.67 |
| 12 | main road | 3 | 1 | 1 | 5 | 1.67 |
| 13 | main road | 3 | 1 | 1 | 5 | 1.67 |
| 14 | main road | 3 | 2 | 1 | 6 | 2.00 |
| 15 | main road | 3 | 2 | 1 | 6 | 2.00 |
| 16 | main road | 3 | 2 | 1 | 6 | 2.00 |
| 17 | secondary road | 3 | 2 | 1 | 6 | 2.00 |
| 18 | secondary road | 3 | 2 | 1 | 6 | 2.00 |
| 19 | main road | 3 | 2 | 1 | 6 | 2.00 |
| 20 | main road | 3 | 3 | 1 | 7 | 2.33 |
| 21 | main road | 3 | 2 | 1 | 6 | 2.00 |
| 22 | main road | 3 | 1 | 1 | 5 | 1.67 |
| 23 | secondary road | 3 | 1 | 1 | 5 | 1.67 |
| 24 | main road | 3 | 1 | 1 | 5 | 1.67 |
| 25 | main road | 3 | 1 | 1 | 5 | 1.67 |
| 26 | main road | 3 | 2 | 1 | 6 | 2.00 |
| 27 | main road | 3 | 3 | 1 | 7 | 2.33 |
| 28 | main road | 3 | 2 | 1 | 6 | 2.00 |
| 29 | main road | 3 | 1 | 1 | 5 | 1.67 |
| 30 | main road | 3 | 1 | 1 | 5 | 1.67 |
| 31 | main road | 3 | 1 | 1 | 5 | 1.67 |
| 32 | main road | 3 | 1 | 1 | 5 | 1.67 |
| 33 | main road | 3 | 2 | 1 | 6 | 2.00 |
| 34 | main road | 3 | 1 | 1 | 5 | 1.67 |
| 35 | main road | 3 | 1 | 1 | 5 | 1.67 |
| 36 | main road | 3 | 1 | 1 | 5 | 1.67 |
| 37 | main road | 3 | 2 | 1 | 6 | 2.00 |
| 38 | main road | 3 | 2 | 1 | 6 | 2.00 |
| 39 | main road | 3 | 2 | 1 | 6 | 2.00 |
| 40 | main road | 3 | 1 | 1 | 5 | 1.67 |
| 41 | main road | 3 | 2 | 1 | 6 | 2.00 |
| 42 | main road | 3 | 2 | 1 | 6 | 2.00 |

| OID | Heliport | transferability | accessibility | e_interdependency | total | mean |
| --- | --- | --- | --- | --- | --- | --- |
| 1 | Vulcanello_heliport | 1 | 1 | 1 | 3 | 1 |
| 4 | LaFossa_heliport | 1 | 3 | 1 | 5 | 1.67 |
| 5 | Piano_heliport | 1 | 1 | 1 | 3 | 1 |

| OID | Harbour | transferability | accessibility | e_interdependency | total | mean |
| --- | --- | --- | --- | --- | --- | --- |
| 2 | PortoPonente_harbour | 2 | 2 | 1 | 5 | 1.67 |
| 3 | PortoLevante_harbour | 2 | 1 | 1 | 4 | 1.33 |
| 6 | Gelso_harbour | 2 | 3 | 1 | 6 | 2 |

**Table S3. Systemic vulnerability**
